# DNA methylation variations and epigenetic aging in telomere biology disorders

**DOI:** 10.1038/s41598-023-34922-1

**Published:** 2023-05-16

**Authors:** Olivia Carlund, Anna Norberg, Pia Osterman, Mattias Landfors, Sofie Degerman, Magnus Hultdin

**Affiliations:** 1 Department of Medical Biosciences, Pathology, Umeå University, Umeå, Sweden; 2 Department of Medical Biosciences, Medical and Clinical Genetics, Umeå University, Umeå, Sweden; 3 Department of Clinical Microbiology, Umeå University, Umeå, Sweden

**Keywords:** Telomeres, DNA methylation, Ageing, Senescence

## Abstract

Telomere Biology Disorders (TBDs) are characterized by mutations in telomere-related genes leading to short telomeres and premature aging but with no strict correlation between telomere length and disease severity. Epigenetic alterations are also markers of aging and we aimed to evaluate whether DNA methylation (DNAm) could be part of the pathogenesis of TBDs. In blood from 35 TBD cases, genome-wide DNAm were analyzed and the cases were grouped based on relative telomere length (RTL): short (S), with RTL close to normal controls, and extremely short (ES). TBD cases had increased epigenetic age and DNAm alterations were most prominent in the ES-RTL group. Thus, the differentially methylated (DM) CpG sites could be markers of short telomeres but could also be one of the mechanisms contributing to disease phenotype since DNAm alterations were observed in symptomatic, but not asymptomatic, cases with S-RTL. Furthermore, two or more DM-CpGs were identified in four genes previously linked to TBD or telomere length (*PRDM8, SMC4, VARS,* and *WNT6)* and in three genes that were novel in telomere biology (*MAS1L*, *NAV2,* and *TM4FS1)*. The DM-CpGs in these genes could be markers of aging in hematological cells, but they could also be of relevance for the progression of TBD.

## Introduction

Telomeres are repetitive sequences at the ends of chromosomes that shorten over time due to insufficient replication of the 3′ end. Critically short telomeres can induce senescence and/or apoptosis to protect the chromosomal integrity^1–3^. Some highly proliferating cells, such as stem cells, germ cells, and activated lymphocytes, delay senescence by activating the telomerase enzyme and thereby re-extending the telomeres. Also, cellular immortalization requires telomere maintenance, usually by upregulating telomerase activity^2,4^. The telomerase enzyme complex consists of the catalytic reverse-transcriptase component (TERT), the RNA-template component (TERC), and several accessory proteins, e. g. dyskerin (encoded by *DKC1*). The telomeres are also associated with a group of proteins, known as the shelterin complex, responsible for capping the telomeres^3^. Short telomere length has been associated with a number of health factors and mortality. Dysfunction of components in either the telomerase or shelterin complexes might result in insufficiency to maintain the telomeric regions and lead to telomere dysfunction and genomic instability^5–9^. Telomere biology disorders (TBDs) are a spectrum of rare conditions characterized by mutations in telomere-related genes. These mutations can lead to pathological telomere shortening and cause a variety of symptoms resembling those of natural aging, including hair greying, degenerative organ failure, and a cancer-prone state. Additionally, TBD patients often have symptoms of bone marrow failure, pulmonary fibrosis, and liver cirrhosis^8,10–12^. Remarkably, some TBD patients have clinical symptoms without critically short telomeres^13^, while other individuals may have critically short telomeres even though they are asymptomatic. It is unclear why telomere length (TL) is not strictly correlated to symptoms in TBD, but this suggests that other factors than telomere attrition are involved in disease progression.

In addition to telomere attrition, epigenetic changes in DNA, RNA, and histones are crucial for regulating senescence and the replicative capacity of cells^14,15^. DNA methylation (DNAm) is a powerful epigenetic mechanism, characterized by the addition of a methyl group to a cytosine-phosphate-guanine (CpG) site. The CpG sites are dispersed throughout the genome and altered CpG DNAm profiles have been identified in cancer, neurological diseases, immunological diseases, atherosclerosis, and osteoporosis^16^. DNAm and TL are independent predictors of aging, health, and mortality^17,18^, but there is an association between altered DNAm and TL^19–21^. Methylation-based profiles could potentially be used to estimate TL in epidemiological studies, although DNAm is not applicable for the analysis of TL in individual cases^21^. DNAm profiles can also be used to estimate epigenetic age with so-called “epigenetic clock models”, which show a high correlation with chronological age^22^. Several studies have indicated that an increased epigenetic age may be linked to cancer risk, all-cause mortality, and neurological disorders^22–26^. Increased epigenetic age has also been suggested in TBD (Dyskeratosis Congenita) and the premature-aging disorders Hutchinson-Gilford Progeria, and Werner syndrome^27–29^, and these disorders also have additional DNAm alterations^30–33^. However, genome-wide DNAm has so far only been analyzed in a few cases of TBDs^30^.

Mutations in telomere-related genes do not seem to fully explain the relationship between TL and the severity of symptoms in TBD, indicating that other mechanisms contribute to the disease phenotype. At present, there are few studies about DNAm alterations in TBD patients, and no genome-wide analysis has been performed in a larger TBD cohort. In this study, we aimed to explore whether genome-wide and gene-specific DNAm profiles could be associated with the phenotypic differences observed in patients with TBD, and to analyze the ongoing epigenetic processes in cells approaching cellular senescence. We also aimed to analyze if premature aging in TBD blood cells affects epigenetic aging. If DNAm alterations are relevant for the disease progression in TBD, drugs affecting DNAm could be a therapeutic option for TBD patients.

## Materials and methods

### Patients and controls

The molecular analysis was conducted in leukocytes from whole blood. Cases with TBD were obtained from the Department of Clinical Genetics, Umeå University Hospital, Sweden, where genetic testing of telomere-related genes and relative telomere length (RTL) measurements have been analyzed in Nordic patients with suspected TBD since 2011. There were 54 samples available from TBD cases with a pathogenic genetic variant, and of those, cases between 16 and 80 years, with no leukemia, and with a sufficient amount of DNA were selected (n = 46). Control samples (n = 20, 19–71 years) were collected from healthy blood donors at Umeå University Hospital.

**Figure 1:**
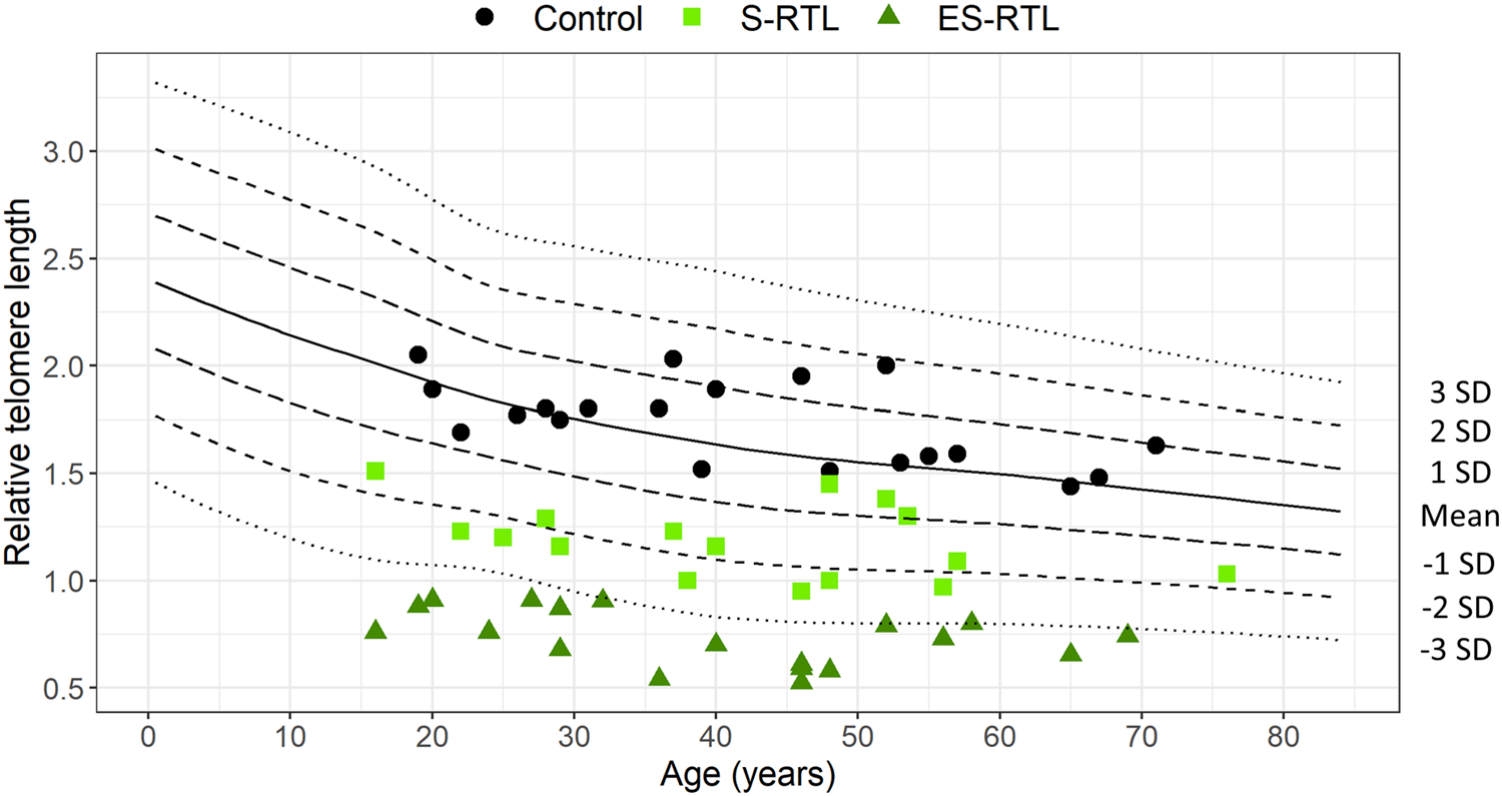
Relative telomere length (RTL) in Telomere Biology Disorder (TBD) cases and controls. RTL versus chronological age in TBD cases with Short (S) RTL (n = 16, light green boxes), Extremely short (ES) RTL (n = 19, dark green triangles), and age-matched controls (n = 20, black circles). The mean value and standard deviations (SD) were calculated from a separate cohort of 174 healthy controls (0–84 years).

The standardized residuals (S_res_) of RTL were calculated with respect to age for the TBD cases and controls, using a separate telomere length control cohort (n = 174, 0–84 years) from the Department of Clinical Genetics as a reference. To analyze potential differences in DNAm in cases with close to normal RTL and extremely short RTL we selected the cases with the largest and the least S_res_. Two distinct groups were chosen: TBD cases with S_res_ > − 2.5 (n = 16) and TBD cases with S_res_ ≤ − 3 (n = 19), resulting in a total of 35 cases included in the study. The cases with S_res_ > − 2.5 were called the Short (S) RTL group and the cases with S_res_ ≤ − 3 the Extremely short (ES) RTL group (Fig. 1, Supplementary Fig. S1a and Table S1).

The 35 TBD cases were probands and relatives from 18 families with a telomere-related mutation in *DKC1* (one individual), *TERC* (nine families, 22 individuals), and *TERT* (eight families, 12 individuals). Phenotypic information was obtained from referrals and classified as hematological (TBD with at least one hematological symptom, e.g. anemia, leukopenia, thrombocytopenia, or hypoplasia), non-hematological (no recorded hematological symptoms, but TBD-associated symptoms such as pulmonary fibrosis, liver cirrhosis, skin/nail/hair changes, etc.), or asymptomatic phenotypes. Among the selected TBD cases, 17 individuals had hematological (H-TBD) symptoms, seven had non-hematological (NH-TBD) symptoms and 11 were asymptomatic (A-TBD) (Supplementary Fig. S1b and Table S1).

The study was approved by the Regional ethical review board in Umeå (Dnr 2016/258-31). All individuals gave their informed consent and the study was conducted in accordance with the Declaration of Helsinki.

### Relative telomere length measurements

Genomic DNA from the TBD cases was extracted by standard protocols at the Department of Clinical Genetics, Umeå University Hospital, Sweden (n = 19) or by Clinical Genetic laboratories in Helsinki, Finland (n = 1), Lund, Sweden (n = 1), Oulu, Finland (n = 4), Stockholm, Sweden (n = 2), Uppsala, Sweden (n = 7), and Århus, Denmark (n = 1). Genomic DNA from the controls was extracted using the Gentra Puregene DNA extraction kit (Gentra Puregene DNA extraction kit, Qiagen, Hilden, DE). DNA concentration and quality were measured with the DeNovix DS-11 (DeNovix, Wilmington, DE, US) instrument for both TBD cases and controls.

RTL was measured by the quantitative-PCR method described by Cawthon^34^ with some minor modifications, as previously described^6^. In short, each DNA sample was measured in triplicates in separate telomere (T) and single-copy gene (S) reactions. A T/S value was calculated for all samples (${2}^{-{(Ct}_{T}-{Ct}_{S})}$, where Ct stands for cycle threshold) and divided by the T/S value of a reference cell line (CCRF-CEM), included in all plates analyzed. The efficiency of the PCR reaction was controlled by including a standard curve of the reference cell line DNA in every run. All DNA samples were analyzed in two separate runs, with the mean value stated as RTL. The results of the qPCR were highly reproducible and the inter-assay coefficient of variation (CV) between the two runs was 2.7 ± 2.2%.

### DNA methylation array analysis

DNA (750–1000 ng) was bisulfite converted using the EZ DNA Methylation Kit (Zymo Research, Irvine, CA), according to the manufacturer's protocol. Efficient bisulfite conversion was confirmed by Methylight *ALU* PCR with the ALU-C4 primer/probe set as previously described^35^. Genome-wide assessment of DNAm profiles was performed using the Infinium MethylationEPIC BeadChip arrays (Illumina, San Diego, CA) with coverage of > 850 000 CpG sites. The raw data were imported into R v4.0.4 and converted to β-values using the Minfi package^36^. The β-value (methylation level) of each CpG site, ranging from 0 (unmethylated) to 1 (fully methylated), was normalized with BMIQ to adjust for the two different probe designs used by the EPIC array^37^. Data pre-processing has been described previously^38^ and includes omitting CpGs with a detection p-value > 0.05, located ≤ 5 bp from a known SNP (single nucleotide polymorphism)^39^, mQTLs (methylation quantitative trait loci)^40,41^, non-specific probes aligning to multiple loci, and duplicated CpGs mapping to the same gene. Gender-related methylation biases were avoided by omitting CpGs located on the X and Y chromosomes (Supplementary Fig. S2). Downstream analysis of methylation data was performed by the Genome Studio software v2011.1 (Illumina) and R statistical software v4.0.4 (R Core Team).

### Adjusting β-values for estimated leukocyte cell composition

Blood cell subtypes have cell-specific DNAm patterns and leukocyte cell composition (LCC) can confound the interpretation of methylation studies in whole blood^42^. Thus, the data set was adjusted for LCC before analysis, where LCC was estimated using the FlowSorted.Blood.EPIC package^43^. DNAm data from sorted leukocytes were obtained from the Gene Expression Omnibus repository (GSE103541) and were normalized and pre-processed in the same way as the TBD cases and controls. The adjusted β-values for TBDs and controls were generated by summarizing the estimated proportion of each cell type multiplied by the mean β- value of the corresponding sorted leukocyte cell type for each CpG.

$${\beta }_{j}={\sum }_{i}^{n}{Estimated\,cell\,proportion}_{i} \times {mean\,\beta\,sorted\,cells}_{ij}$$

where *j* is the selected CpG site and *i* is the cell type (CD4 T-cells, CD8 T-cells, B-cells, monocytes, and granulocytes). The mean β-value for each CpG site was calculated for the TBD groups (ES-RTL, S-RTL, H-TBD, NH-TBD, and A-TBD) and then subtracted from the controls. The threshold for differentially methylated (DM) CpG sites was set to |Δβ|≥ 0.2.

### Statistical analysis

Statistical analysis was performed in R v4.0.4. The Kruskal–Wallis test, followed by Dunn’s test and Bonferroni correction, was used for comparing groups. Fischer’s exact test followed by Bonferroni correction was used to compare the distribution of DM-CpGs on each chromosome. A heatmap was created by the ComplexHeatmap package^44^. Epigenetic age was estimated with Horvath’s pan-tissue clock^22^, the PhenoAge clock^23^, and the epiTOC clock^24^, and the epigenetic ages were then adjusted for chronological age and estimated granulocyte proportion. Gene ontology analysis was performed with the GeneGO MetaCore™ software (Thomson Reuters, New York, NY) using the enrichment analysis workflow tool. The analysis was based on genes with DM-CpGs with |Δβ|≥ 0.2 and the results were presented as gene ontology (GO) processes by the MetaCore software. Epigenetic regulation of gene expression pathways was retrieved from the Reactome pathway knowledgebase (Epigenetic regulation of gene expression (Homo sapiens), R-HSA-212165)^45^.

## Results

### Relative telomere length in telomere biology disorder cases and controls

The study included 35 TBD cases in 18 families, all with a previously identified telomere-related mutation in *DKC1* (n = 1), *TERC* (n = 22), or *TERT* (n = 12), and 20 age-matched controls (Supplementary Table S1). The TBD cases were selected based on the standardized residuals (S_res_) of the relative telomere length (RTL) with respect to age. The RTL values were considered short in 16 cases (S-RTL; 16–76 years; S_res_ > -2.5), and extremely short in 19 cases (ES-RTL; 16–69 years; S_res_ ≤ -3) (Fig. 1, Supplementary Fig. S1a and Table S1). There was a significant difference in RTL between the groups, with mean RTL values of 1.74 ± 0.19 (controls), 1.18 ± 0.17 (S-RTL), and 0.73 ± 0.13 (ES-RTL) (p < 0.001). There was no significant difference in chronological age between the groups, with mean ages of 42.1 ± 16.0 (controls), 42.0 ± 15.7 (S-RTL), and 39.9 ± 15.9 (ES-RTL) (p = 0.888) (Table 1). In total, 17 cases had hematological symptoms (H-TBD), seven had non-hematological symptoms (NH-TBD) and 11 were asymptomatic (A-TBD) (Supplementary Fig. S1b and Table S1). The RTL values were significantly shorter in all phenotypic groups compared to controls (p < 0.001) but there was no significant difference in chronological age between any of the groups (p = 0.283) (Supplementary Table S2). RTL and clinical symptoms varied within the families, even among individuals carrying the same mutation.

**Table 1:** Age, relative telomere length (RTL), and epigenetic age in cases based on RTL and controls. Mean values and standard deviations for chronological age, RTL, standardized residuals (S_res_), ΔepiTOC (mitotic age), ΔPhenoAge (phenotypic age), and ΔHorvath’s pan-tissue age for Short (S) RTL, Extremely short (ES) RTL, and controls (C). Statistical analysis was performed by the Kruskal–Wallis test followed by Dunn’s test with Bonferroni correction.

	Controls (n = 20)	Short RTL (n = 16)	Extremely short RTL (n = 19)	p-value
Chronological age (years)	42.1 ± 16.0	42.0 ± 15.7	39.9 ± 15.9	0.888
Relative telomere length (RTL)	1.74 ± 0.19	1.18 ± 0.17	0.73 ± 0.13	< 0.001^1^
Standardized residuals (S_res_) of RTL	0.33 ± 0.66	− 1.81 ± 0.63	− 3.60 ± 0.39	< 0.001^2^
ΔepiTOC	0 ± 0.006	0.011 ± 0.019	0.018 ± 0.026	0.008^3^
ΔPhenoAge	0 ± 2.4	6.3 ± 9.0	10.2 ± 9.9	< 0.001^4^
ΔHorvath’s pan-tissue	0 ± 3.4	− 3.0 ± 5.6	− 1.5 ± 10.0	0.112

^1^S-RTL versus C p = 0.003, ES-RTL versus C p < 0.001, ES-RTL versus S-RTL p = 0.003.

^2^S-RTL versus C p = 0.003, ES-RTL versus C p < 0.001, ES-RTL versus S-RTL p = 0.004.

^3^S-RTL versus C p = 0.055, ES-RTL versus C p = 0.011, ES-RTL versus S-RTL p = 1.000.

^4^S-RTL versus C p = 0.044, ES-RTL versus C p < 0.001, ES-RTL versus S-RTL p = 0.476.

### Differential DNA methylation in TBD cases

**Figure 2:**
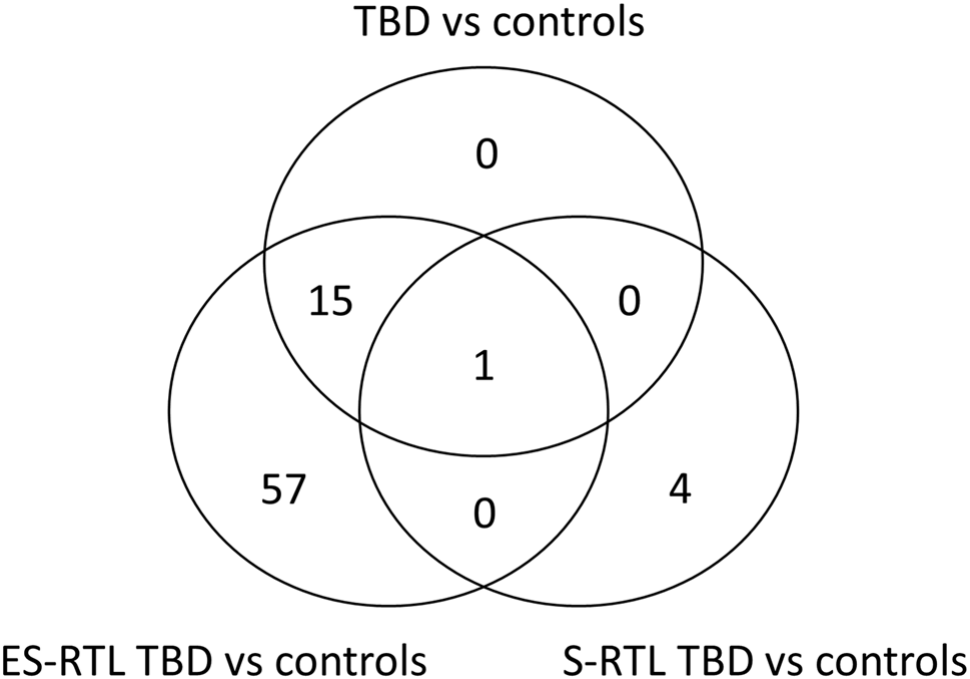
Differently methylated (DM) CpG sites in Telomere Biology Disorder (TBD) cases compared to controls. Venn diagram showing overlapping DM-CpGs between all TBD cases and controls, between cases with Extremely short (ES) relative telomere length (RTL) and controls, and between cases with Short (S) RTL and controls.

Genome-wide DNAm profiling was performed using the Infinium MethylationEPIC BeadChip arrays and all β-values (methylation levels) were adjusted for leukocyte cell composition (LCC). A principal component analysis (PCA) of all CpGs (n = 689 959) could not separate the data into groups based on RTL or symptoms (Supplementary Fig. S3). The mean β-value for each CpG was calculated for controls, S-RTL, and ES-RTL, and a CpG was considered differentially methylated (DM) if the difference in DNAm between groups exceeded |Δβ|≥ 0.2. With this approach, 77 DM-CpGs mapping to 48 genes were identified (Fig. 2). Seventy-three DM-CpGs mapping to 45 genes were identified in the ES-RTL group compared to controls, and five DM-CpGs mapping to four genes were identified in the S-RTL group compared to controls. Of the 73 DM-CpGs identified in the ES-RTL group, 72 were unique to the group and one overlapped with the five sites identified in S-RTL. Only two DM-CpGs were identified when comparing the S-RTL TBDs with the ES-RTL TBDs. Comparing all 35 TBD cases to controls identified 16 DM-CpGs, all overlapping with the DM-CpGs in the ES-RTL group. Since the majority of DM-CpGs were identified in the ES-RTL group, we focused the analysis on those 73 DM-CpGs.

**Figure 3:**
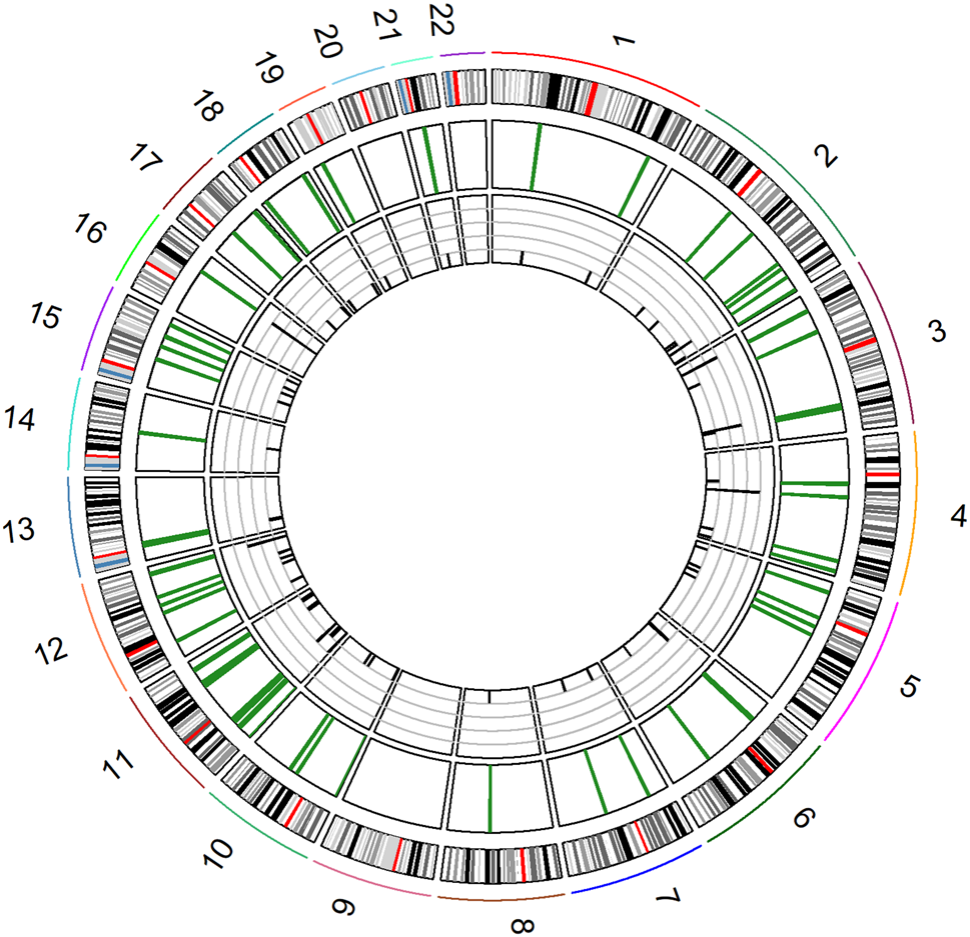
Ideogram mapping of differentially methylated (DM) CpGs in the Extremely short relative telomere length (ES-RTL) group. Chromosomal distribution of the 73 DM-CpGs found in Telomere Biology Disorder (TBD) cases with ES-RTL compared to controls. The outer circle represents the autosomal chromosomes 1–22, where the red line marks each centromeric region. The green lines of the middle circle represent the location of DM-CpGs. The height of the black vertical lines of the inner circle represents the number of DM-CpGs at each chromosomal location, represented by the grey horizontal lines (n = 1–5).

The majority of the 73 DM-CpGs were associated with coding genes (70% of the hypomethylated DM-CpGs and 71% of the hypermethylated DM-CpGs) and the remaining DM-CpGs were located in intergenic regions. The DM-CpGs were mainly located in low CpG-dense regions (70% of the hypomethylated DM-CpGs and 63% of the hypermethylated DM-CpGs) (Supplementary Fig. S4). Ideogram mapping of the DM-CpGs was performed to evaluate the specific distribution among all 22 chromosomes included in the analysis. The sites were dispersed throughout 19 chromosomes and Fischer’s exact test showed that DM-CpGs were underrepresented on chromosome 1 (p = 0.045) and overrepresented on chromosomes 4 and 11 (p = 0.039 and p = 0.010, respectively). However, after Bonferroni correction there was no significant difference between the dispersion of DM-CpGs at any chromosome (Fig. 3, Supplementary Table S3).

**Figure 4:**
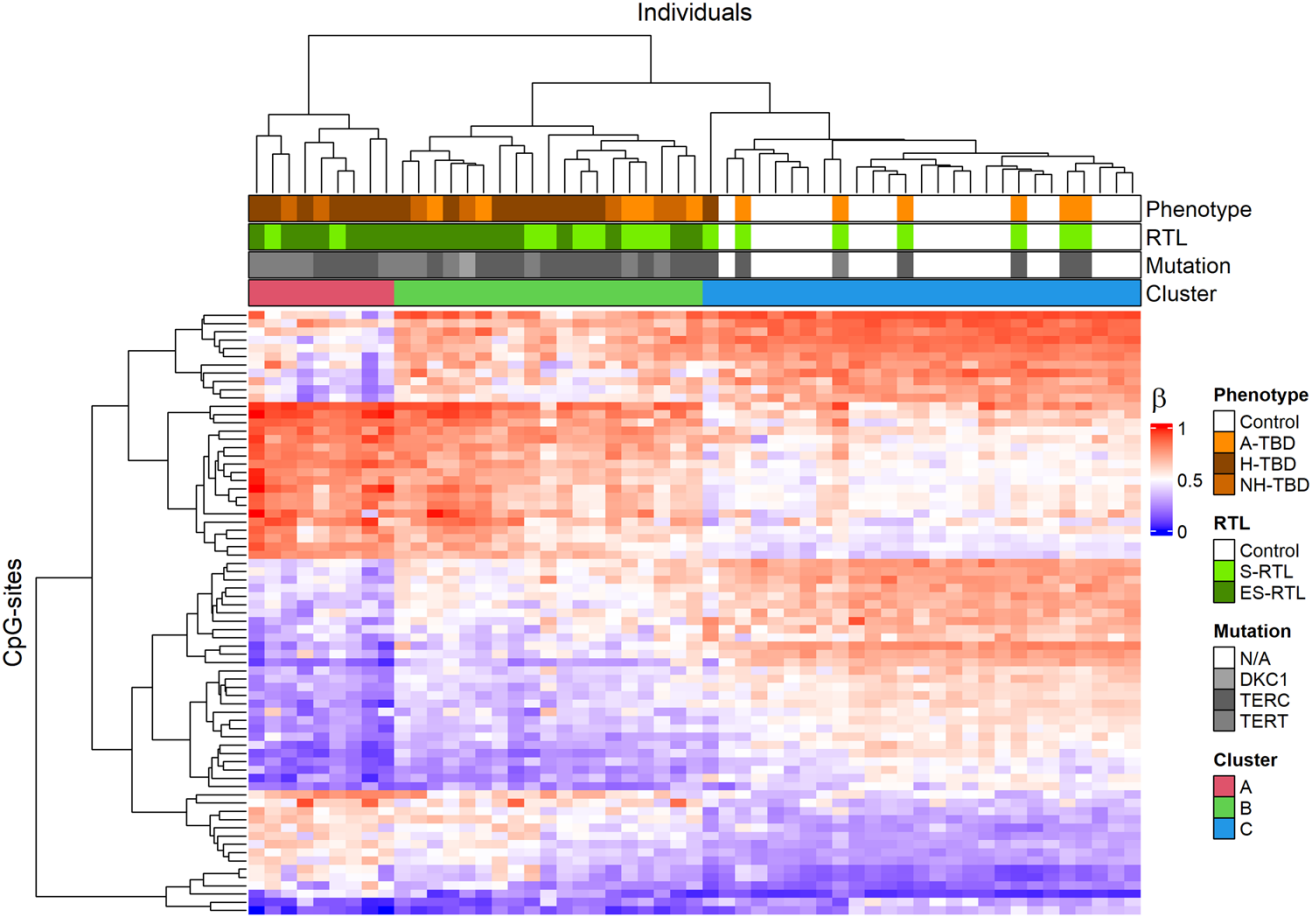
Cluster analysis of differentially methylated (DM) CpGs in the Extremely short relative telomere length (ES-RTL) group. Heatmap clustering of the 73 DM-CpGs found in Telomere Biology Disorder (TBD) cases with ES-RTL. Rows correspond to CpG sites (n = 73) and columns to individuals (n = 55). The methylation level is represented by a β- value between 0–1 where 0 is completely unmethylated (blue) and 1 is fully methylated (red). Bars below the top dendrogram give information about phenotype group, RTL group, mutation status, and cluster abbreviation. A-TBD = Asymptomatic TBD, H-TBD = Hematological TBD, NH-TBD = Non-hematological TBD, S-RTL = Short RTL. N/A = no mutation.

Hierarchical clustering analysis of the 73 DM-CpGs in the ES-RTL group identified three distinct clusters: A, B, and C (Fig. 4). Cluster A consisted of nine TBD cases, mainly with ES-RTL (7 cases) and/or H-TBD (7 cases). Cluster B varied more in phenotypic presentation and consisted of 19 TBD cases, 12 of which belonged to the ES-RTL group. Cluster C consisted of all the controls and seven cases with S-RTL (six asymptomatic and one H-TBD). In total, there were three asymptomatic cases with ES-RTL and none of these were identified in cluster C. These data show that some asymptomatic cases have altered DNAm patterns compared to controls, although they are phenotypically normal. Only a few DM-CpGs (n = 5) were found when comparing the S-RTL group to controls, indicating that the methylation profiles of these groups are relatively similar. However, almost all symptomatic S-RTL cases clustered with the ES-RTL cases instead of controls.

The 73 DM-CpGs were not associated with genes commonly involved in TBD or other suggested disorders affecting telomeres^7^, and not in genes associated with epigenetic regulation of gene expression^45^. Gene ontology analysis, performed with the GeneGO MetaCore software, showed that the genes with DM-CpGs were involved in developmental processes (animal organ, nervous system, system, head, multicellular organism, adipose tissue, and brain), differentiation (fat cell and cell), and cellular developmental processes (Supplementary Table S4). Methylation profiles of the *TERC* and *TERT* genes did not differ between cases with/without said mutation (n = 22 mutated in *TER*C and n = 12 mutated in *TERT*) or between cases and controls (Supplementary Fig. S5).

### Genes with multiple DM-CpGs in TBD cases with Extremely short RTL

To avoid potential random genes with single DM-CpGs, only genes that had two or more DM-CpGs were further examined. In the ES-RTL group, five genes fulfilled the criteria: *MAS1L*, *PRDM8, SMC4*, *VARS*, and *WNT6* (Table 2). Hypermethylated sites were located in the 1^st^ Exon of *MAS1L* (cg12423733, cg25730428), the TSS200 and 5'UTR of *PRDM8* (cg19409579, cg27018912, cg05059566), the gene body of *VARS* (cg17619755, cg08899667), and the gene body and 3'UTR of *WNT6* (cg00011225, cg13903421). Three hypomethylated sites were located in the gene body of *SMC4* (cg14322760, cg01464849, cg26663696). None of these sites were differentially methylated in the S-RTL group.

**Table 2:** Genes with ≥ 2 differentially methylated (DM) CpGs in cases with Extremely short relative telomere length (ES-RTL). The annotations are from the Infinium MethylationEPIC BeadChip manifest (Illumina, San Diego, CA).

Gene name	CpG site	Map location (GRCh37)	Genetic location	Relation to CpG island	Δβ in ES-RTL
*MAS1L*	cg12423733 cg25730428	29454623 29454755	1stExon 1stExon	Open Sea Open Sea	0.211 0.206
*PRDM8*	cg19409579 cg27018912 cg05059566	81118500 81118602 81118647	TSS200; 5’UTR TSS200; 5’UTR TSS200; 5’UTR	Island Island Shore	0.206 0.202 0.216
*SMC4*	cg14322760 cg01464849 cg26663696	160120464 160120481 160121275	Body Body Body	Shore Shore Shelf	-0.253 -0.227 -0.267
*VARS*	cg17619755 cg08899667	31760629 31761055	Body Body	Shelf Shelf	0.249 0.219
*WNT6*	cg00011225 cg13903421	219738314 219738714	Body 3’UTR	Island Island	0.204 0.206

### DNA methylation in TBD cases associated with different phenotypes

Only a few DM-CpGs (n = 5) were identified between S-RTL and controls. However, cluster analysis showed that symptomatic and asymptomatic S-RTL cases were located in different groups (Fig. 4). Comparing all A-TBDs (n = 11) with controls identified only 4 DM-CpGs with a |Δβ|≥ 0.2. This indicated that the DNAm profiles of A-TBD cases at group level were very similar to controls and hence might conceal methylation differences in the symptomatic S-RTL group. Indeed, comparing only the symptomatic S-RTL cases (n = 8) with controls identified 42 DM-CpGs, mapping to 28 genes. Of those DM-CpGs, 23 sites (55%) overlapped with the 73 DM-CpGs identified in the ES-RTL group which could explain the outcome of the cluster analysis. Genes with more than two DM-CpGs in the symptomatic S-RTL group were identified in *NAV2* (cg01282852, cg03026982, cg10473623), *SMC4 (*cg14322760, cg01464849, cg26663696), and *WNT6 (*cg22587479, cg00011225, cg13903421, cg25242471) (Supplementary Table S5).

Cluster analysis and separation of the S-RTL group into symptomatic and asymptomatic cases showed that the methylation differences were more pronounced in cases with symptoms. However, it could not be excluded that the cases with H-TBD (n = 17), with phenotype related to insufficient hematopoiesis, could have different DNAm patterns in blood compared to cases with NH-TBD (n = 7). Thus, the analysis was extended to evaluate if methylation differences could be observed in cases with H-TBD and NH-TBD compared to controls. In total, 122 DM-CpGs mapping to 89 genes were identified in the different phenotypic groups (Supplementary Fig. S6). Of these DM-CpGs, 64 (52%) overlapped with the 73 DM-CpGs identified in the ES-RTL group, which could be expected since the majority of symptomatic cases had ES-RTL. Genes that were differentially methylated at two or more CpGs were identified in H-TBD; *NAV2* (cg01282852, cg03026982, cg10473623), *PRDM8* (cg19409579, cg27018912, cg05059566, cg02458885), *SMC4* (cg14322760, cg01464849, cg26663696), *TM4FS1* (cg10725542, cg26584465), *VARS* (cg17619755, cg08899667), and *WNT6* (cg22587479, cg00011225, cg13903421, cg25242471) and in NH-TBD: *SMC4 (*cg14322760, cg01464849, cg26663696) and *WNT6 (*cg22587479, cg00011225, cg13903421, cg25242471) (Supplementary Table S6).

Gene ontology analysis, performed with the GeneGO MetaCore software, showed that the 89 genes with DM-CpGs identified in the phenotypic groups were involved in cell-adhesion processes (cell, cell–cell, cell–cell adhesion via plasma-membrane adhesion molecules, and homophilic cell adhesion via plasma membrane adhesion molecules), animal organ development, detection (of other organism, and of external biotic stimulus), positive regulation of interferon-gamma production, cerebellar granule cell differentiation, and cerebellar granular layer formation (Supplementary Table S7).

### Epigenetic aging in TBD cases

**Figure 5:**
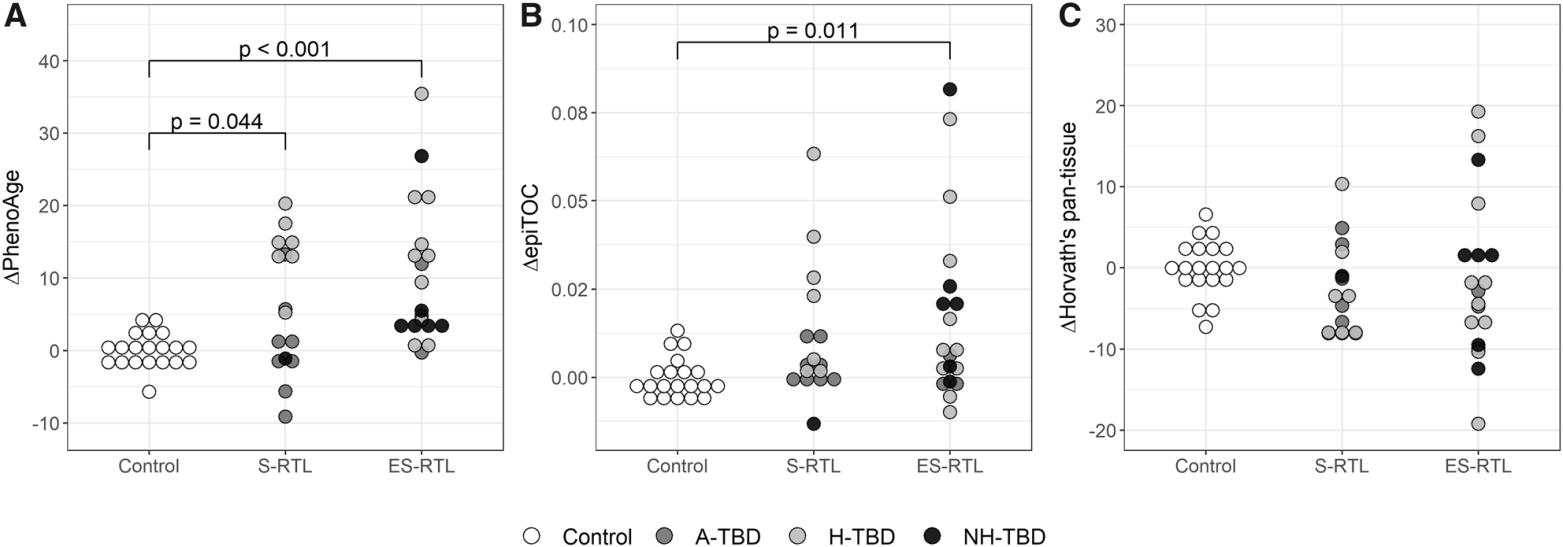
Epigenetic age in the relative telomere length (RTL) groups. The y-axis shows delta epigenetic age and the x-axis the compared groups (controls (white circles), Short (S) RTL cases, and Extremely short (ES) RTL cases). Phenotype groups are represented by color. A-TBD = Asymptomatic Telomere Biology Disorder (dark gray), H-TBD = Hematological TBD (light gray), and NH-TBD = Non-hematological TBD (black). (**A**) The PhenoAge clock shows a significant difference between ES-RTL and controls (p < 0.001) and S-RTL and controls (p = 0.044). (**B**) The mitotic clock (epiTOC) shows a significant difference between ES-RTL and controls (p = 0.011). (**C**) No significant difference between any group using Horvath’s pan-tissue clock (p = 0.112).

Epigenetic age in TBDs and controls was estimated with three epigenetic clock models: Horvath’s pan-tissue clock, the PhenoAge clock, and the epiTOC clock, all based on age-specific DNAm alterations^22–24^. The CpGs defining each clock model did not overlap with the DM-CpGs identified in TBD (n = 77 in S-RTL and ES-RTL, n = 122 in A-TBD, H-TBD, and NH-TBD). The models estimated the epigenetic age for each case and control, and the epigenetic age was then adjusted for chronological age and granulocyte proportion. Increased phenotypic age (ΔPhenoAge) was found in both the S-RTL group (6.3 ± 9.0) and the ES-RTL group (10.2 ± 9.9) compared to controls (0 ± 2.4), (p = 0.044 and p < 0.001, respectively) (Table 1, Fig. 5a) while mitotic age (ΔepiTOC) was increased in the ES-RTL (0.018 ± 0.026) group compared to controls (0 ± 0.006) (p = 0.011) (Table 1, Fig. 5b). For the S-RTL group, there was a trend towards increased mitotic age (0.011 ± 0.019) compared to controls, although not significant (p = 0.055). There was no difference in epigenetic age between any of the groups using Horvath’s pan-tissue clock (p = 0.112) (Table 1, Fig. 5c).

For the phenotypic TBD groups, increased phenotypic age (ΔPhenoAge) was found in H-TBDs (13.7 ± 8.5) compared to A-TBDs (1.8 ± 6.8) and controls (0 ± 2.4) (p = 0.004 and p < 0.001, respectively) (Supplementary Table S2 and Fig. S7a). Mitotic age (ΔepiTOC) was increased in H-TBDs (0.020 ± 0.025) compared to controls (0 ± 0.006) (p = 0.005) (Supplementary Table S2 and Fig. S7b). No difference was observed between any of the groups using Horvath’s pan-tissue clock (p = 0.117) (Supplementary Table S2 and Fig. S7c).

## Discussion

TBDs are a heterogeneous group of diseases, characterized by defective telomerase function or telomere maintenance. The patients present with a wide range of symptoms, from asymptomatic or mild anemia to severe bone marrow failure, idiopathic pulmonary fibrosis, and/or premature death^7,12,46^. In general, very short telomeres are associated with more severe symptoms and an earlier age of onset. However, the symptoms of the patients are not strictly correlated to telomere length, even in patients carrying the same mutation^13^. In the present study, we aimed to evaluate whether epigenetic changes genome-wide and in specific genes could be a mechanism explaining the phenotypic differences found in patients with TBD. We also aimed to analyze the patterns of epigenetic aging in TBDs and to evaluate if the premature telomere-related cellular aging also was reflected in age-associated DNAm changes.

All TBD cases had RTL below the mean value of normal controls. To study the differences in DNAm among TBD cases and between TBDs and controls, we selected cases with the largest and the least standardized residual RTL with respect to age. The cases with S-RTL often had RTL values that could be seen in control samples. However, some S-RTL cases within the normal range had telomere-related symptoms. One limitation with the RTL measurements, and hence the classification into S-RTL and ES-RTL, were that some of the samples were extracted by different centers. Some studies have indicated that different DNA extraction methods may contribute to potential variations which could influence RTL measurements^47–49^. However, in this study, TBD cases with short telomeres were analyzed and the potential effect of different protocols could only have a minor effect for a few cases. Furthermore, as none of the cases with standardized residuals between -2.5 and -3 were included, the risk of changing RTL group due to potential extraction method differences was reduced.

When comparing the ES-RTL, the S-RTL, and the control group, the largest difference in DNAm was observed between ES-RTL and controls. Only a few DM-CpG sites were identified when comparing the two RTL groups against each other or the S-RTL with controls, suggesting that the differences between these groups were not so pronounced. However, potential differences among individual cases can be concealed as the analysis was made on group level. The definition set to classify a CpG site as differentially methylated (|Δβ|≥ 0.2) was rather strict and may reduce the sensitivity for potential CpG sites of interest, but was chosen in order to increase the specificity of the results. A limitation with the methylation arrays was that they do not provide full coverage of CpG sites. A future perspective to further explore the biological relevance of DNAm for TBD is to perform whole genome bisulfite sequencing for full resolution of CpG site methylation which also will allow for conclusive differently methylated region analysis^50^. The DM-CpGs in the ES-RTL group were enriched in gene bodies and were not chromosome-specific. Previous studies have suggested that proximity to short telomeres could be of importance for the development of the DM-CpGs^32,51^. However, the number of identified DM-CpGs in our cohort was not sufficient to test this hypothesis.

Cluster analysis of the 73 DM-CpGs in the ES-RTL group compared to controls identified distinct patterns of methylation changes. In the S-RTL group, half of the cases were asymptomatic and the other half were symptomatic (mainly H-TBD). At group level, there were only minor differences in DM-CpGs between the S-RTL cases and the controls or the ES-RTL cases. However, the symptomatic S-RTL cases behaved as the ES-RTL group in the cluster analysis, and the majority of asymptomatic S-RTL cases were found together with the controls. These results indicated that the heterogeneity concerning symptoms in the S-RTL group could affect the outcome of the differential methylation analysis. Support for that hypothesis was found when DM-CpGs were analyzed in the symptomatic S-RTL cases. Although having RTL close to normal controls, these cases had a methylation profile reminding of the ES-RTL group. These results indicated that the identified DM-CpGs could be of importance for the development of the symptomatic phenotype in cases not having extremely short telomere lengths. In TBD, loss of hematopoietic stem cells and progenitors causes bone marrow failure leading to cytopenias^7,12^. DNAm was analyzed in leukocytes and in NH-TBD and A-TBD the blood and bone marrow showed no sign of disease. In H-TBD, where the tissue of the analyzed cells showed signs of disease, more DM-CpGs were found than in NH-TBD or A-TBD. Thus, the DM-CpGs found in symptomatic TBD and the corresponding genes could be involved in the disease process. However, we cannot exclude that the DM-CpGs are markers for subpopulations of hematological cells with better potential for proliferation that will have a selection advantage and be more prevalent in an exhausted stem cell pool.

All asymptomatic TBDs with ES-RTL clustered with the symptomatic TBD cases and not with the controls. This was not surprising since the cluster analysis was based on the DM-CpGs found in the ES-RTL group. However, this indicates that extensive telomere shortening could generate altered DNAm profiles, even in asymptomatic cases. Support for this was the altered DNAm observed in ES-RTL cases with NH-TBD, i.e. with no reported hematological symptoms. An alternative hypothesis is that the DNAm profiles in asymptomatic TBD with ES-RTL show that the cases are in transition toward symptom development. However, from our cohort it was difficult to conclude whether DNAm changes in TBD were mainly driven by telomere length or phenotype or if they were a combination of both. In future studies, it could be of interest to follow A-TBD cases and analyze if methylation changes could be used to predict the disease progress.

Methylation analyses in the H-TBD and NH-TBD groups and the symptomatic cases in the S-RTL group showed a high overlap with DM-CpGs in the ES-RTL group. Not surprisingly, there was also a high degree of overlap of genes with two or more DM-CpGs between these groups. All four groups had two or more DM-CpGs in the *SMC4* and *WNT6* genes. In addition to those, three genes (*MAS1L*, *PRDM8,* and *VARS*) fulfilled the criteria in the ES-RTL group, four genes (*NAV2, PRDM8, TM4FS1,* and *VARS*) in H-TBDs, and one gene (*NAV2*) in the symptomatic S-RTL group. For some of the identified genes, there are previous correlations to leukocyte telomere length (LTL) and TBD supporting a possible role in the disease progression. The *SMC4* gene encodes a protein involved in chromosome condensation and has recently been associated with both LTL and telomere dysfunction^52,53^. In Werner syndrome, transcriptional downregulation and DM-CpGs have been observed in *SMC4*^31^, but the potential implication of DNAm changes in *SMC4* in relation to TBD is so far unknown. The *WNT6* gene encodes a protein involved in several developmental processes. Interestingly, the WNT signaling pathway can be downregulated by dysfunctional telomeres in Dyskeratosis Congenita, and restoration of WNT signaling has been shown to increase the expression of the shelterin protein complex and recap telomeres^54,55^. DNA-methylation has been described as a mechanism to regulate *WNT* and *WNT6* expression. Hypermethylation of three of the *WNT6* CpGs that we identified (cg06795233, cg22587479, cg00011225) has been associated with increased *WNT6* expression in glioma^56^. If *WNT* hypermethylation is present at an early stage in subpopulations of stem cells, these cells could have an advantage if the telomeres get critically short. Alternatively, short telomeres could be a signal to increase methylation of *WNT* and thereby improve the telomere capping. In future studies of TBD patients, it would be of interest to analyze if hypermethylation of these CpGs might affect *WNT6* expression. If so, perhaps WNT agonists could be used to reduce symptoms in TBD. In the patients with non-hematological symptoms (NH-TBD), only *SMC4* and *WNT6* had two or more DM-CpGs. This could indicate that DM-CpGs in these genes in leukocytes are early events in TBD and preside any future overt hematological symptoms. The genes with DM-CpGs observed in the other groups could have more correlation to events in the hematological stem cells in TBD-induced insufficient hematopoiesis. However, the limited number of NH-TBD cases could also have affected the outcome of that analysis.

Two genes (*PRDM8* and *VARS*) had two or more DM-CpGs in both the ES-RTL and H-TBD groups. The *PRDM8* gene encodes a histone methyltransferase of importance for hematopoietic and neuronal differentiation in induced pluripotent stem cells^57^. Aberrant DNAm in *PRDM8* has also been observed previously in the premature aging disorders Dyskeratosis Congenita, Down syndrome, Hutchinson-Gilford Progeria syndrome, and Werner syndrome, but it is unknown if DM-CpGs in *PRDM8* are involved in the disease processes or merely act as a biomarker for cellular aging^30,31,57^. The *VARS* gene encodes Valyl-tRNA synthetase and has been correlated to LTL^19^. Several CpG sites in *VARS* have been described among the 30 most significant sites associated with LTL in normal blood. If the methylation status of *VARS* is a marker for short LTL, the DM-CpGs found in our study can be associated with the short telomeres and not the disease process of the TBD. Future studies will have to show if the *VARS* gene is of relevance in TBD.

The *NAV2* gene was differentially methylated in H-TBDs and symptomatic S-RTL cases. As almost all cases in the symptomatic S-RTL group were H-TBDs, the DM-CpGs in *NAV2* could be related to hematological symptoms. The *NAV2* gene encodes a protein involved in cellular growth and migration, and no association with telomere length or TBD has previously been shown^58^. The *MAS1L* gene was differentially methylated in the ES-RTL group. The role of this gene, encoding a proto-oncogene-like G protein-coupled receptor^59^, is unknown and has no previous correlation to telomeres. Finally, the *TM4FS1* gene was differentially methylated among the H-TBDs. The TM4FS1 protein mediates signal transduction events and is involved in the regulation of cell development, growth, mobility, and activation^60^. However, no previous studies have shown any association between *TM4FS1* and telomere biology.

Gene ontology analysis showed that the genes with DM-CpGs were involved in developmental and differentiation processes, and for the phenotypic TBDs also cell adhesion, interferon-gamma production, and detection of organisms. No correlation to epigenetic or telomere-related processes was found. However, as DNAm is a regulator of gene expression, a hypothesis could be that DM-CpGs in *TERT* or *TERC* could be a mechanism to increase telomerase activity among TBD cases. Mutations in the *TERT* promoter and DNAm of the *TERT* promoter including the *TERT* Hypermethylated Oncological Region (THOR) have been suggested as a mechanism to increase telomerase activity^61^. Furthermore, DM-CpGs in *TERC* have been associated with altered *TERC* expression in patients with breast cancer^62^. However, our study did not find any DM-CpGs in *TERT* or *TERC*, suggesting that this was not a mechanism to increase gene expression of these genes among the TBD cases. These data were in concordance with a previous study where DNAm in *TERT* and *TERC* were analyzed in patients with Dyskeratosis Congenita^30^. Still, a potential limitation is that the arrays do not cover all the CpGs in *TERT* and *TERC*.

There are several models based on DNA methylation that can be used to estimate the epigenetic age, mitotic age, and phenotypic age of different tissues. In contrast to chronological aging, the pace of epigenetic aging varies among individuals and may predict the health span or onset of disease in presymptomatic carriers^22–24^. In this study, no differences in epigenetic age were found using Horvath’s pan-tissue clock, which is in concordance with a previous study of TBD^30^. Horvath’s pan-tissue clock is highly correlated to chronological age and does not capture cell replication or cellular senescence, which could explain why no difference was observed between the TBD cases and controls. The epiTOC clock (mitotic age) focuses on CpGs in polycomb group target promoter regions, where DNAm levels increase with mitosis^24^. The PhenoAge clock is based on divergence in the physiological rate of aging and not on chronological age. Increased PhenoAge has been associated with increased mortality risk among individuals of the same age^23^. In our study, analysis of the epiTOC and PhenoAge clocks showed that Δage was increased in both the ES-RTL and S-RTL groups compared to controls. One plausible explanation is that the telomere attrition causing premature aging also is reflected in the PhenoAge model and shown as an older phenotype. Furthermore, the increased mitotic age could reflect an increased demand of the hematological stem cell pool. A decreased telomere length could promote stem cell exhaustion, forcing the remaining stem cell pool to divide more frequently to maintain the homeostasis of hematological cells, and consequently increase the ΔepiTOC age. Support for that hypothesis could be the results when analyzing Δage in the different phenotypic groups (H-TBD, NH-TBD, and A- TBD). H-TBD showed an increased ΔepiTOC age compared to controls, as well as an increased ΔPhenoAge compared to A-TBD and controls. These results could potentially reinforce the hypothesis that premature aging and increased cell division among remaining hematological stem cells contribute to the changes in the leukocyte CpGs included in the PhenoAge and epiTOC clocks. None of the CpG sites used to identify epigenetic age for Horvath’s pan-tissue-, PhenoAge-, and epiTOC clocks overlapped with our identified DM-CpG sites with |Δβ|≥ 0.2. Thus, the changes in ΔPhenoAge and ΔepiTOC age are most likely not related to our DM-CpGs but rather to processes involving other sites displaying the epigenetic age. Accordingly, the observation that ΔPhenoAge was increased in the S-RTL group compared to controls could also support the hypothesis that epigenetic events are an early sign in the development of TBD.

In summary, our study showed that the TBD cases had an increased epigenetic age compared to controls, which is potentially a sign of premature cellular aging and higher demand on the stem cells. At group level, cases with symptoms showed altered DNAm profiles compared to controls. Of special interest were the patterns observed in the symptomatic cases with RTL close to normal controls, indicating that methylation changes could be a part of disease progression. Interestingly, cluster analysis showed that most asymptomatic cases were grouped with controls, but with symptomatic cases if they had extremely short telomeres. The biology behind the differences between these asymptomatic cases remains elusive and requires further investigation. Furthermore, the functional relevance of the differently methylated CpGs identified in seven genes (*MAS1L*, *NAV2, PRDM8, SMC4*, *TM4FS1, VARS*, and *WNT6)*, for gene expression, telomere maintenance, and progression of disease in TBDs needs to be explored in future studies.

## Supplementary Information

Supplementary Information 1.

Supplementary Figure 1.

Supplementary Figure 2.

Supplementary Figure 3.

Supplementary Figure 4.

Supplementary Figure 5.

Supplementary Figure 6.

Supplementary Figure 7.

## Data Availability

The dataset generated (GSE224847) during the current study is available in the Gene Expression Omnibus repository, https://www.ncbi.nlm.nih.gov/geo/.
