# Supplementary material for: DNA methylation variations and epigenetic aging in telomere biology disorders
Source: Sci Rep. 2023 May 16;13:7955. doi: 10.1038/s41598-023-34922-1 (PMC10188573; doi:10.1038/s41598-023-34922-1)
Supplement: Supplementary file 1 — Supplementary Information 1. [file 41598_2023_34922_MOESM1_ESM.docx]

# DNA Methylation Variations and Epigenetic Aging in Telomere Biology Disorders

Olivia Carlund^1^, Anna Norberg^2^, Pia Osterman^1^, Mattias Landfors^1^, Sofie Degerman^1,3^, Magnus Hultdin^1^*

^1^ Department of Medical Biosciences, Pathology, Umeå University, Umeå, Sweden

^2^ Department of Medical Biosciences, Medical and Clinical Genetics, Umeå University, Umeå, Sweden

^3^ Department of Clinical Microbiology, Umeå University, Umeå, Sweden

* Corresponding author: magnus.hultdin@umu.se

# Supplementary files

## Supplementary tables

**Telomere Biology Disorder (TBD) cases and control characteristics.**

| Individual | Age | RTL | Standardized residual (S_res_) | Mutation | Phenotype |
| --- | --- | --- | --- | --- | --- |
| Control | 36 | 1.80 | +0.45 | N/A | N/A |
| Control | 40 | 1.89 | +0.95 | N/A | N/A |
| Control | 67 | 1.48 | +0.15 | N/A | N/A |
| Control | 22 | 1.69 | -0.68 | N/A | N/A |
| Control | 52 | 2.00 | +1.86 | N/A | N/A |
| Control | 20 | 1.89 | -0.11 | N/A | N/A |
| Control | 57 | 1.59 | +0.32 | N/A | N/A |
| Control | 28 | 1.80 | +0.08 | N/A | N/A |
| Control | 53 | 1.55 | +0.06 | N/A | N/A |
| Control | 48 | 1.51 | -0.22 | N/A | N/A |
| Control | 37 | 2.03 | +1.34 | N/A | N/A |
| Control | 65 | 1.44 | -0.10 | N/A | N/A |
| Control | 55 | 1.58 | +0.23 | N/A | N/A |
| Control | 71 | 1.63 | +0.99 | N/A | N/A |
| Control | 19 | 2.05 | +0.35 | N/A | N/A |
| Control | 29 | 1.75 | -0.06 | N/A | N/A |
| Control | 31 | 1.80 | +0.23 | N/A | N/A |
| Control | 26 | 1.77 | -0.15 | N/A | N/A |
| Control | 46 | 1.95 | +1.42 | N/A | N/A |
| Control | 39 | 1.52 | -0.47 | N/A | N/A |
| Short RTL | 40 | 1.16 | -1.77 | *TERC* | Hematological |
| Short RTL | 52 | 1.38 | -0.65 | *TERT* | Asymptomatic |
| Short RTL | 16 | 1.51 | -1.64 | *TERT* | Hematological |
| Short RTL | 46 | 0.95 | -2.43 | *TERC* | Asymptomatic |
| Short RTL | 48 | 1.00 | -2.22 | *TERC* | Hematological |
| Short RTL | 48 | 1.45 | -0.47 | *TERC* | Hematological |
| Short RTL | 56 | 0.97 | -2.29 | *TERT* | Hematological |
| Short RTL | 54 | 1.30 | -0.96 | *TERC* | Hematological |
| Short RTL | 57 | 1.09 | -1.78 | *TERC* | Asymptomatic |
| Short RTL | 25 | 1.20 | -2.40 | *TERC* | Asymptomatic |
| Short RTL | 22 | 1.23 | -2.34 | *TERC* | Asymptomatic |
| Short RTL | 38 | 1.00 | -2.44 | *TERC* | Hematological |
| Short RTL | 37 | 1.23 | -1.64 | *TERT* | Asymptomatic |
| Short RTL | 28 | 1.29 | -1.88 | *TERC* | Asymptomatic |
| Short RTL | 76 | 1.03 | -1.70 | *TERT* | Non-hematological |
| Short RTL | 29 | 1.16 | -2.28 | *TERC* | Asymptomatic |
| Extremely short RTL | 36 | 0.54 | -4.21 | *TERT* | Hematological |
| Extremely short RTL | 46 | 0.61 | -3.73 | *TERC* | Hematological |
| Extremely short RTL | 46 | 0.59 | -3.81 | *TERT* | Hematological |
| Extremely short RTL | 65 | 0.65 | -3.61 | *TERC* | Hematological |
| Extremely short RTL | 46 | 0.52 | -4.06 | *TERT* | Hematological |
| Extremely short RTL | 52 | 0.79 | -3.05 | *TERC* | Hematological |
| Extremely short RTL | 19 | 0.88 | -3.57 | *TERC* | Hematological |
| Extremely short RTL | 24 | 0.76 | -4.08 | *DKC1* | Non-hematological |
| Extremely short RTL | 69 | 0.74 | -3.18 | *TERC* | Non-hematological |
| Extremely short RTL | 29 | 0.68 | -4.09 | *TERT* | Non-hematological |
| Extremely short RTL | 20 | 0.91 | -3.43 | *TERC* | Asymptomatic |
| Extremely short RTL | 27 | 0.91 | -3.39 | *TERC* | Asymptomatic |
| Extremely short RTL | 58 | 0.80 | -3.02 | *TERC* | Non-hematological |
| Extremely short RTL | 56 | 0.73 | -3.29 | *TERT* | Hematological |
| Extremely short RTL | 29 | 0.87 | -3.37 | *TERC* | Hematological |
| Extremely short RTL | 16 | 0.76 | -4.09 | *TERT* | Hematological |
| Extremely short RTL | 48 | 0.58 | -3.86 | *TERC* | Non-hematological |
| Extremely short RTL | 32 | 0.91 | -3.06 | *TERC* | Asymptomatic |
| Extremely short RTL | 40 | 0.70 | -3.48 | *TERT* | Non-hematological |

**Table S1.** Individual, age (years), relative telomere length (RTL), standardized residual (S_res_), mutation, and phenotype in Telomere Biology Disorders (TBD) cases and controls**.Age, relative telomere length, and epigenetic age in cases based on phenotype and controls.**

|  | Controls  (n=20) | Asymptomatic TBD (n=11) | Hematological TBD (n=17) | Non-hematological TBD (n=7) | p-value |
| --- | --- | --- | --- | --- | --- |
| Chronological age (years) | 42.1 ± 16.0 | 34.1 ± 12.4 | 41.8 ± 14.6 | 49.1 ± 19.7 | 0.283 |
| Relative telomere length (RTL) | 1.74 ± 0.19 | 1.11 ± 0.17 | 0.90 ± 0.31 | 0.76 ± 0.14 | <0.001^1^ |
| Standardized residuals (S_res_) of RTL | 0.33 ± 0.66 | -2.30 ± 0.82 | -2.86 ± 1.14 | -3.34 ± 0.84 | <0.001^2^ |
| ΔepiTOC | 0 ± 0.006 | 0.003 ± 0.005 | 0.020 ± 0.025 | 0.020 ± 0.031 | 0.005^3^ |
| ΔPhenoAge | 0 ± 2.4 | 1.8 ± 6.8 | 13.7 ± 8.5 | 6.2 ± 9.3 | <0.001^4^ |
| ΔHorvath’s pan-tissue | 0 ± 3.4 | -4.2 ± 4.8 | -1.5 ± 9.9 | -0.7 ± 8.5 | 0.117 |

^1^ A-TBD vs C p=0.005, H-TBD vs C p <0.001, NH-TBD vs C p <0.001, H-TBD vs A-TBD p=0.964, NH-TBD vs A-TBD p=0.416, NH-TBD vs H-TBD p=1.000

^2^ A-TBD- vs C p=0.001, H-TBD vs C p <0.001, NH-TBD vs C p <0.001, H-TBD vs A-TBD p=1.000, NH-TBD vs A-TBD p=1.000, NH-TBD vs H-TBD p=1.000

^3^ A-TBD vs C p=1.000, H-TBD vs C p=0.005, NH-TBD vs C p=0.154, H-TBD vs A-TBD p=0.475, NH-TBD vs A-TBD p=1.000, NH-TBD vs H-TBD p=1.000

^4^A-TBD vs C p=1.000, H-TBD vs C p<0.001, NH-TBD vs C p=0.198, H-TBD vs A-TBD p=0.004, NH-TBD vs A-TBD p=1.000, NH-TBD vs H-TBD p=0.722

**Table S2.** Mean values and standard deviations for chronological age, relative telomere length (RTL), standardized residuals (S_res_), ΔepiTOC (mitotic age), ΔPhenoAge, and ΔHorvath’s pan-tissue age, for asymptomatic (A) Telomere Biology Disorder (TBD), hematological (H) TBD, non-hematological (NH) TBD, and controls (C). Statistical analysis was performed by the Kruskal-Wallis test followed by Dunn’s test with Bonferroni correction.

**Chromosomal distribution of the differentially methylated CpGs in the Extremely short relative telomere length group.**

| Chromosome | Number of DM-CpGs | Number of non-DM-CpGs | Odds ratio | P-value | Adjusted  p-value |
| --- | --- | --- | --- | --- | --- |
| 1 | 2 | 67190 | 0.26 | 0.045 | 1.000 |
| 2 | 7 | 53317 | 1.27 | 0.509 | 1.000 |
| 3 | 8 | 40945 | 1.95 | 0.079 | 1.000 |
| 4 | 7 | 29972 | 2.34 | 0.039 | 0.863 |
| 5 | 4 | 36750 | 1.03 | 0.796 | 1.000 |
| 6 | 5 | 43809 | 1.08 | 0.809 | 1.000 |
| 7 | 2 | 37058 | 0.50 | 0.440 | 1.000 |
| 8 | 1 | 31187 | 0.29 | 0.265 | 1.000 |
| 9 | 0 | 21768 | 0.00 | 0.177 | 1.000 |
| 10 | 3 | 33868 | 0.83 | 1.000 | 1.000 |
| 11 | 10 | 40497 | 2.55 | 0.010 | 0.223 |
| 12 | 7 | 37019 | 1.87 | 0.114 | 1.000 |
| 13 | 2 | 16841 | 1.13 | 0.699 | 1.000 |
| 14 | 1 | 24406 | 0.38 | 0.524 | 1.000 |
| 15 | 4 | 23313 | 1.66 | 0.315 | 1.000 |
| 16 | 4 | 30235 | 1.26 | 0.563 | 1.000 |
| 17 | 2 | 36087 | 0.51 | 0.593 | 1.000 |
| 18 | 2 | 12518 | 1.52 | 0.383 | 1.000 |
| 19 | 1 | 30686 | 0.30 | 0.383 | 1.000 |
| 20 | 0 | 19432 | 0.00 | 0.275 | 1.000 |
| 21 | 1 | 8355 | 1.13 | 0.589 | 1.000 |
| 22 | 0 | 14633 | 0.00 | 0.411 | 1.000 |

**Table S3.** Chromosomal distribution of the differentially methylated (DM) CpGs (n=73) identified in the Extremely short relative telomere length (ES-RTL) group. Statistical analysis was performed with Fisher’s exact test followed by Bonferroni correction.

**Enrichment analysis by gene ontology processes in the Extremely short relative telomere length group.**

| Number | GO processes | Total genes in GO process | P-value | FDR | Total genes in the ES-RTL data set | Network objects from the ES-RTL data set |
| --- | --- | --- | --- | --- | --- | --- |
| 1 | animal organ development | 5201 | 4.355E-07 | 1.330E-03 | 24 | Myosin I, CACNA1D, PTPR-mu, Leptin receptor, ARID5B, NAV2, MYO1E, WNT, ITGA2, Galpha(q)-specific Class A Orphan/other GPCRs, CREB5, SOX5, ATF/CREB, HMGA2, Lphn3, WNT6, CDK6, PLC-beta, Prdm8, Palladin, SOCS3, CACNA1 L-type, LEC2, GAS2 |
| 2 | fat cell differentiation | 198 | 1.018E-06 | 1.395E-03 | 6 | ARID5B, WNT, CREB5, ATF/CREB, HMGA2, PLC-beta |
| 3 | cell differentiation | 5188 | 1.946E-06 | 1.395E-03 | 23 | Myosin I, PTPR-mu, Leptin receptor, MKP-3, ARID5B, PIWIL4, NAV2, MYO1E, WNT, ITGA2, NCAM2, CREB5, SOX5, ATF/CREB, HMGA2, Lphn3, WNT6, CDK6, PLC-beta, Prdm8, Palladin, SOCS3, CACNA1 L-type |
| 4 | cellular developmental process | 5224 | 2.205E-06 | 1.395E-03 | 23 | Myosin I, PTPR-mu, Leptin receptor, MKP-3, ARID5B, PIWIL4, NAV2, MYO1E, WNT, ITGA2, NCAM2, CREB5, SOX5, ATF/CREB, HMGA2, Lphn3, WNT6, CDK6, PLC-beta, Prdm8, Palladin, SOCS3, CACNA1 L-type |
| 5 | nervous system development | 3256 | 2.283E-06 | 1.395E-03 | 18 | Myosin I, PTPR-mu, Leptin receptor, NAV2, WNT, Galpha(q)-specific Class A Orphan/other GPCRs, NCAM2, SOX5, ATF/CREB, HMGA2, Lphn3, WNT6, CDK6, PLC-beta, Prdm8, Palladin, CACNA1 L-type, LEC2 |
| 6 | system development | 3256 | 3.399E-06 | 1.425E-03 | 24 | Myosin I, CACNA1D, PTPR-mu, Leptin receptor, ARID5B, NAV2, MYO1E, WNT, ITGA2, Galpha(q)-specific Class A Orphan/other GPCRs, NCAM2, SOX5, ATF/CREB, HMGA2, Lphn3, WNT6, CDK6, PLC-beta, Prdm8, Palladin, SOCS3, CACNA1 L-type, LEC2, GAS2 |
| 7 | head development | 1457 | 3.718E-06 | 1.425E-03 | 12 | Myosin I, ARID5B, NAV2, WNT, Galpha(q)-specific Class A Orphan/other GPCRs, ATF/CREB, HMGA2, Lphn3, CDK6, PLC-beta, Prdm8, LEC2 |
| 8 | multicellular organism development | 6758 | 3.733E-06 | 1.425E-03 | 26 | Myosin I, CACNA1D, PTPR-mu, Leptin receptor, ARID5B, IFI17, PIWIL4, NAV2, MYO1E, WNT, ITGA2, Galpha(q)-specific Class A Orphan/other GPCRs, NCAM2, SOX5, ATF/CREB, HMGA2, Lphn3, WNT6, CDK6, PLC-beta, Prdm8, Palladin, SOCS3, CACNA1 L-type, LEC2, GAS2 |
| 9 | adipose tissue development | 72 | 6.817E-06 | 2.314E-03 | 4 | ARID5B, CREB5, ATF/CREB, HMGA2 |
| 10 | brain development | 1385 | 1.453E-05 | 3.764E-03 | 11 | Myosin I, NAV2, WNT, Galpha(q)-specific Class A Orphan/other GPCRs, ATF/CREB, HMGA2, Lphn3, CDK6, PLC-beta, Prdm8, LEC2 |

**Table S4**. Enrichment analysis by gene ontology (GO) processes for the differentially methylated genes (n=45) in the Extremely short relative telomere length group (ES-RTL). The analysis was based on Entrez Gene ID and the top ten GO processes are presented. The data was accessed 2022-11-02 from the GeneGO MetaCore™ software (Thomson Reuters, New York, NY).

**Genes with ≥2 differentially methylated CpGs in symptomatic cases with Short relative telomere length.**

| Gene name | CpG site | Map Location (GRCh37) | Genetic location | Relation to CpG island | Δβ in symptomatic S-RTL |
| --- | --- | --- | --- | --- | --- |
| *NAV2* | cg01282852  cg03026982  cg10473623 | 19953170  19953699  20119176 | Body  Body  Body | Open Sea  Open Sea  Open Sea | -0.221  -0.208  -0.209 |
| *SMC4* | cg14322760  cg01464849  cg26663696 | 160120464  160120481  160121275 | Body  Body  Body | Shore  Shore  Shelf | -0.253  -0.203  -0.238 |
| *WNT6* | cg22587479  cg00011225  cg13903421  cg25242471 | 219738226  219738314  219738714  219738732 | Body  Body  3'UTR  3'UTR | Island  Island  Island  Island | 0.208  0.223  0.236  0.216 |

**Table S5**. Genes with two or more differentially methylated (DM) CpG sites in symptomatic cases with Short relative telomere length (S-RTL) The annotations are from the MethylationEPIC BeadChip manifest (Illumina, San Diego, CA).

**Genes with ≥2 differentially methylated CpGs in symptomatic cases.**

| Gene name | CpG site | Map Location (GRCh37) | Genetic location | Relation to CpG island | Δβ in H-TBD | Δβ in NH-TBD |
| --- | --- | --- | --- | --- | --- | --- |
| *NAV2* | cg01282852  cg03026982  cg10473623 | 19953170  19953699  20119176 | Body  Body  Body | Open Sea  Open Sea  Open Sea | -0.242  -0.219  -0.252 |  |
| *PRDM8* | cg19409579  cg27018912  cg05059566  cg02458885 | 81118500  81118602  81118647  81119249 | TSS200; 5’UTR  TSS200; 5’UTR  TSS200; 5’UTR  5’UTR | Island  Island  Shore  Island | 0.219  0.227  0.249  0.204 |  |
| *SMC4* | cg14322760  cg01464849  cg26663696 | 160120464  160120481  160121275 | Body  Body  Body | Shore  Shore  Shelf | -0.271  -0.218  -0.264 | -0.259  -0.243  -0.274 |
| *TM4SF1* | cg10725542  cg26584465 | 149094653  149095006 | Body  Body | Open Sea  Open Sea | -208  -207 |  |
| *VARS* | cg17619755  cg08899667 | 31760629  31761055 | Body  Body | Shelf  Shelf | 0.214  0.203 |  |
| *WNT6* | cg22587479  cg00011225  cg13903421  cg25242471 | 219738226  219738314  219738714  219738732 | Body  Body  3’UTR  3’UTR | Island  Island  Island  Island | 0.225  0.244  0.242  0.225 | 0.207  0.218  0.224  0.213 |

**Table S6**. Genes with two or more differentially methylated (DM) CpG sites in cases with hematological (H) Telomere Biology Disorder (TBD) or non-hematological (NH) TBD. The annotations are from the MethylationEPIC BeadChip manifest (Illumina, San Diego, CA).

**Enrichment analysis by gene ontology processes in the symptomatic cases.**

| Number | GO processes | Total genes in GO process | P-value | FDR | Total genes in the symptomatic data set | Network objects from the symptomatic data set |
| --- | --- | --- | --- | --- | --- | --- |
| 1 | cell-cell adhesion via plasma-membrane adhesion molecules | 357 | 4.833E-15 | 1.599E-11 | 17 | PCDHGA4, PTPR-mu, Protocadherin gamma B1, PCDHGB4, PCDHGB3, PCDHGA8, PCDHGA6, PCDHGB2, Palladin, PCDHGA7, PCDHGA2, L-selectin, PCDHGA3, LEC2, PCDHGA5, Lphn3, PCDHGA1 |
| 2 | homophilic cell adhesion via plasma membrane adhesion molecules | 222 | 3.257E-14 | 5.388E-11 | 14 | PCDHGA4, PTPR-mu, Protocadherin gamma B1, PCDHGB4, PCDHGB3, PCDHGA8, PCDHGA6, PCDHGB2, Palladin, PCDHGA7, PCDHGA2, PCDHGA3, PCDHGA5, PCDHGA1 |
| 3 | cell-cell adhesion | 774 | 1.388E-13 | 1.531E-10 | 21 | PCDHGA4, PTPR-mu, Protocadherin gamma B1, PCDHGB4, PCDHGB3, PCDHGA8, NCAM2, PCDHGA6, PCDHGB2, Palladin, PCDHGA7, PCDHGA2, L-selectin, PCDHGA3, LEC2, Dectin-1, PCDHGA5, WNT, ITGA2, Lphn3, PCDHGA1 |
| 4 | cell adhesion | 1288 | 7.874E-13 | 6.514E-10 | 25 | PCDHGA4, Myosin I, PTPR-mu, Protocadherin gamma B1, PCDHGB4, PCDHGB3, PCDHGA8, NCAM2, PCDHGA6, PCDHGB2, PAR3-beta, Palladin, PCDHGA7, PCDHGA2, L-selectin, PCDHGA3, LEC2, Dectin-1, PCDHGA5, ADAM3, WNT, ITGA2, Lphn3, PLC-beta, PCDHGA1 |
| 5 | animal organ development | 5201 | 2.879E-06 | 1.906E-03 | 37 | LHX6, Myosin I, CACNA1D, PAX5, PTPR-mu, c-Maf, ARID5B, USP2, BAAT, NAV2, SOX5, NFIX, DDRGK1, DNA ligase IV, Palladin, LEC2, Leptin receptor, Rod1, Zeta-sarcoglycan, MMP-20, MYO1E, WNT, ITGA2, Galpha(q)-specific Class A Orphan/other GPCRs, NF-I, MHC class II beta chain, Lphn3, MHC Class I alpha chain, WNT6, CDK6, PLC-beta, Prdm8, HLAC, SOCS3, Matrin-3, CACNA1 L-type, GAS2 |
| 6 | detection of other organism | 39 | 9.726E-06 | 5.167E-03 | 4 | Dectin-1, MHC class II beta chain, MHC Class I alpha chain, HLAC |
| 7 | positive regulation of interferon-gamma production | 145 | 1.093E-05 | 5.167E-03 | 6 | Dectin-1, IL-1RI, HLA-DPB, WNT, MHC class II beta chain, MHC Class I alpha chain |
| 8 | detection of external biotic stimulus | 52 | 3.094E-05 | 1.138E-02 | 4 | Dectin-1, MHC class II beta chain, MHC Class I alpha chain, HLAC |
| 9 | cerebellar granule cell differentiation | 19 | 3.639E-05 | 1.138E-02 | 3 | NFIX, WNT, NF-I |
| 10 | cerebellar granular layer formation | 19 | 3.639E-05 | 1.138E-02 | 3 | NFIX, WNT, NF-I |

**Table S7**. **Enrichment analysis by gene ontology (GO) processes** for the differentially methylated genes (n=89) in the symptomatic Telomere Biology Disorder groups. The analysis was based on Entrez Gene ID and the top ten GO processes are presented. The data was accessed 2022-11-02 from the GeneGO MetaCore™ software (Thomson Reuters, New York, NY).

## Supplementary figure legends

**Figure S1.** **Standardized residuals (S_res_) for Telomere Biology Disorder (TBD) cases and controls.** The black solid line (S_res_= 0) is the age-adjusted mean value of a separate telomere length control cohort (n=174, 0-84 years) used as a reference. The black dashed line (S_res_= -2.5) marks the cutoff for Short (S) relative telomere length (RTL). A) S_res_ versus chronological age in TBD cases with S-RTL (n=16, light green boxes), Extremely short (ES) RTL (n=19, dark green triangles), and age-matched controls (n=20, black circles). B) S_res_ versus chronological age in asymptomatic (A) TBD cases (n=11, light orange diamonds), hematological (H) TBD cases (n=17, brown triangles), non-hematological (NH) TBD cases (n=7, dark orange boxes), and age-matched controls (n=20, black circles).

**Figure S2. Data pre-processing of the Telomere Biology Disorder (TBD) cases and controls.** Scheme of normalization and filtration before differential methylation analysis in TBD cases and controls, performed with R statistical software v4.0.4 (R Core Team).

**Figure S3. Principal component analysis of the methylation data.**

Principal component analysis of all CpGs (n=689 959). Principal component (PC) 1, 2 and 3 explained 14.5%, 11.8%, and 6.5% of the variation, respectively. A) and B) are colored by relative telomere length (RTL) group (control = black, Short (S) RTL = light green, Extremely short (ES) RTL = dark green). C) and D) are colored by symptom group (control = black, asymptomatic telomere biology disorder (A-TBD) = light orange, hematological (H) TBD = brown, non-hematological (NH) TBD = dark orange).

**Figure S4. Location of differentially methylated CpGs in cases with Extremely short relative telomere length.** Hypo- and hypermethylated CpGs in cases with Extremely short (ES) relative telomere length (RTL) and their location in genes (left) and relationship to islands (right). The number of hypo- and hypermethylated CpGs are 41 and 32, respectively. The annotations are from the Infinium MethylationEPIC BeadChip manifest (Illumina, San Diego, CA) based on GRCh37, and here, Promoter = TSS1500 and TSS200, Intragenic = 5’UTR, 1stExon, gene body, and 3’UTR. A few CpGs had double annotations which are included in the figure. CpGs can be located within an island, island shore (shore), island shelf (shelf), or distant from an island region (open sea). A) Distribution of hypomethylated CpGs B) Distribution of hypermethylated CpGs. Note that in the right pie chart, the sum of the percentage is 101 since double annotations are included.

**Figure S5.** **Methylation levels in the *TERC* and *TERT* genes.** The x-axis represents the genomic location (GRCh37) present on the Infinium MethylationEPIC BeadChip arrays after filtration. Promoter region = TSS1500 and TSS200 (black bar), Intragenic region = 5’UTR, 1stExon, gene body, and 3’UTR (grey bar). The y-axis represents the mean β- value for each CpG. A) The *TERC* gene (n=9 CpGs, chr3: 169,482,714-169,483,295). Black circles = CpG, red solid line = individuals with *TERC* mutation (n=22), black dashed line = individuals with *TERT* or *DKC1* mutation (n=13), grey dashed line = controls (n=20). None of the CpGs were differentially methylated (i.e. had a |Δβ|≥0.2) between any of the groups. B) The *TERT* gene (n=88 CpGs, chr5: 1,253,833-1,296,007). Black circles = CpG, red solid line = individuals with *TERT* mutation (n=12), black dashed line = individuals with *TERC* or *DKC1* mutation (n=23), grey dashed line = controls (n=20). None of the CpGs were differentially methylated (i.e. had a |Δβ|≥0.2) between any of the groups.

**Figure S6.** **Differently methylated (DM) CpG sites in Telomere Biology Disorder (TBD) cases compared to controls.** Venn diagram showing overlapping DM-CpGs between asymptomatic (A) TBDs and controls (C), hematological (H) TBDs and controls, and non-hematological (NH) TBDs and controls.

**Figure S7. Epigenetic age in phenotype groups.** The y-axis shows delta epigenetic age and the x-axis the compared groups (controls (white circles), asymptomatic (A) Telomere Biology Disorder (TBD), hematological (H) TBD, and non-hematological (NH) TBD). Relative telomere length (RTL) groups are represented by color. S-RTL = Short RTL (grey), ES-RTL = Extremely short RTL (black). A) The PhenoAge clock shows a significant difference between H-TBD and controls (p<0.001) and H-TBD and A-TBD (p=0.004). B) The mitotic clock (epiTOC) shows a significant difference between H-TBD and controls (p=0.005). C) No significant difference between any group using Horvath’s pan-tissue clock (p=0.117).
