# Supplementary figures and images for: DNA methylation variations and epigenetic aging in telomere biology disorders
Source: Sci Rep. 2023 May 16;13:7955. doi: 10.1038/s41598-023-34922-1 (PMC10188573; doi:10.1038/s41598-023-34922-1)

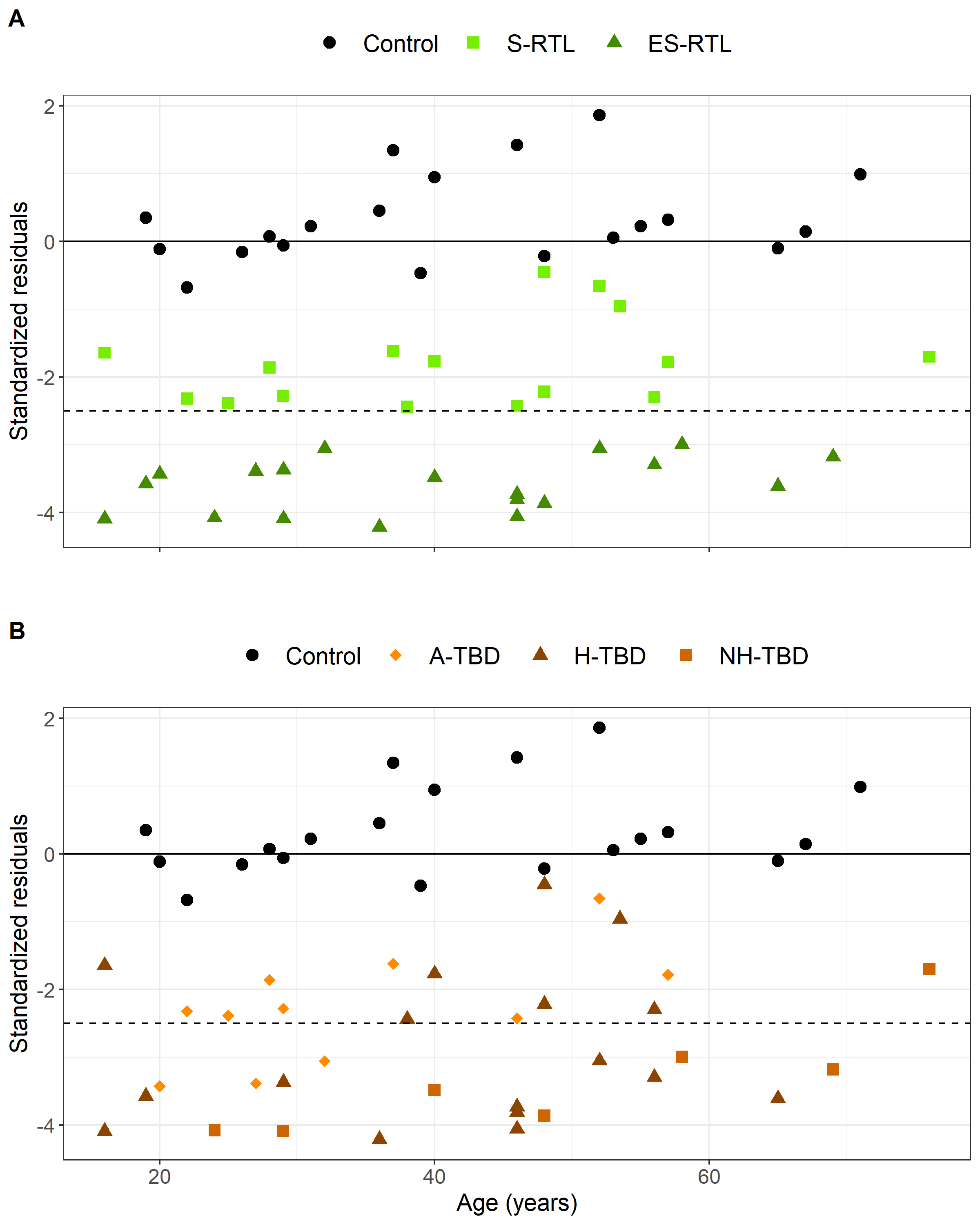

Supplement: Supplementary file 2 — Supplementary Figure 1. [file 41598_2023_34922_MOESM2_ESM.tiff]

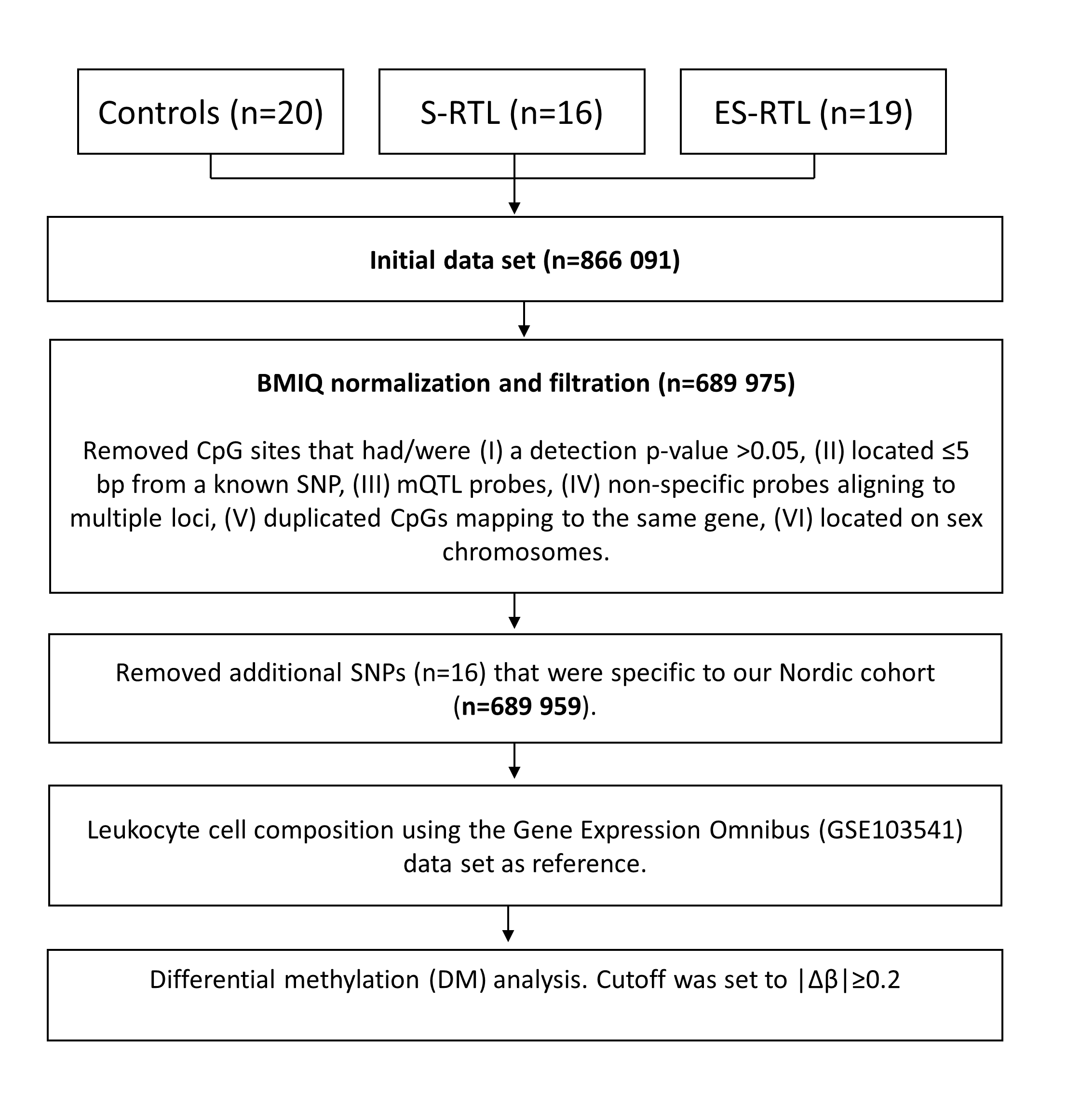

Supplement: Supplementary file 3 — Supplementary Figure 2. [file 41598_2023_34922_MOESM3_ESM.tif]

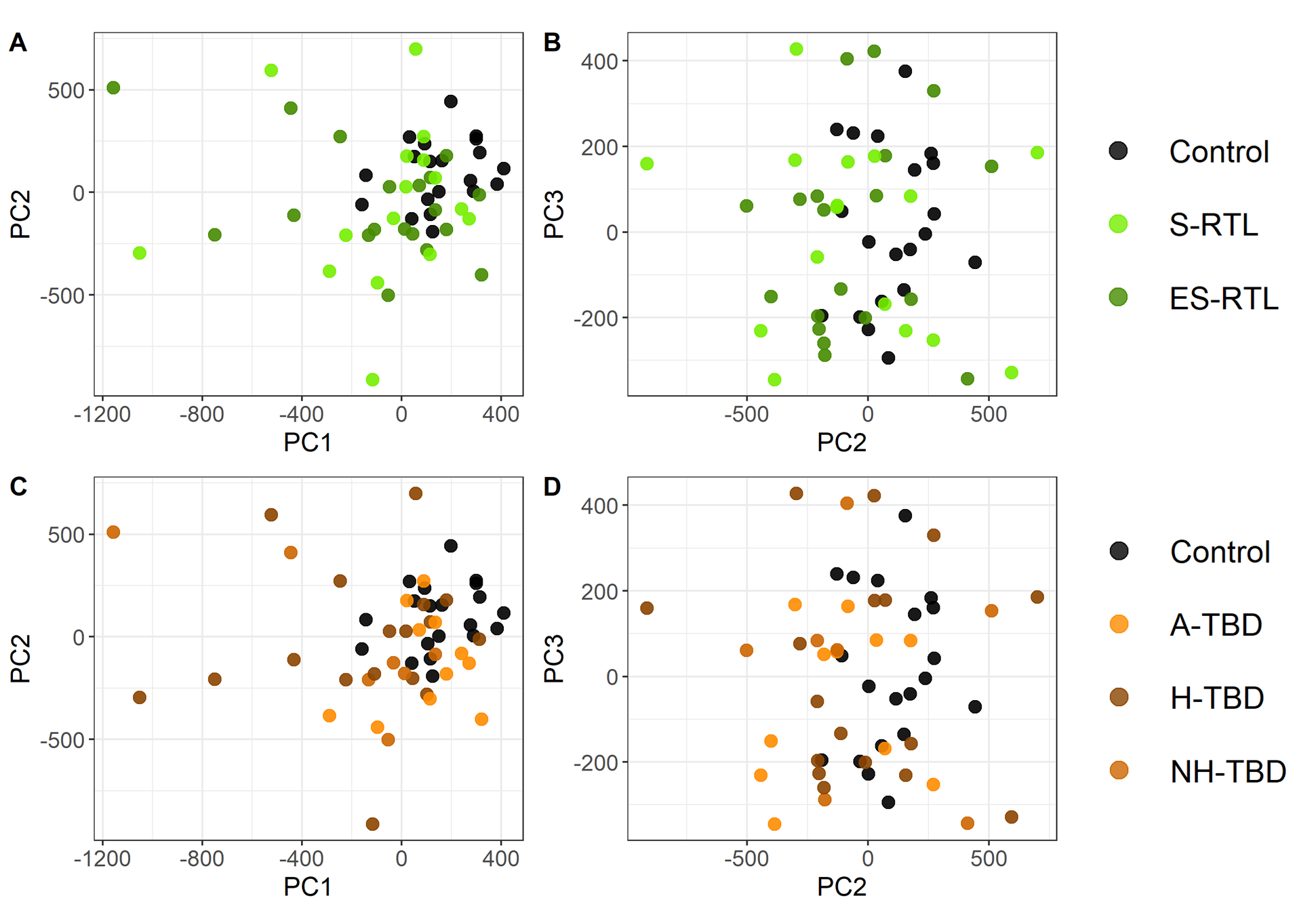

Supplement: Supplementary file 4 — Supplementary Figure 3. [file 41598_2023_34922_MOESM4_ESM.tif]

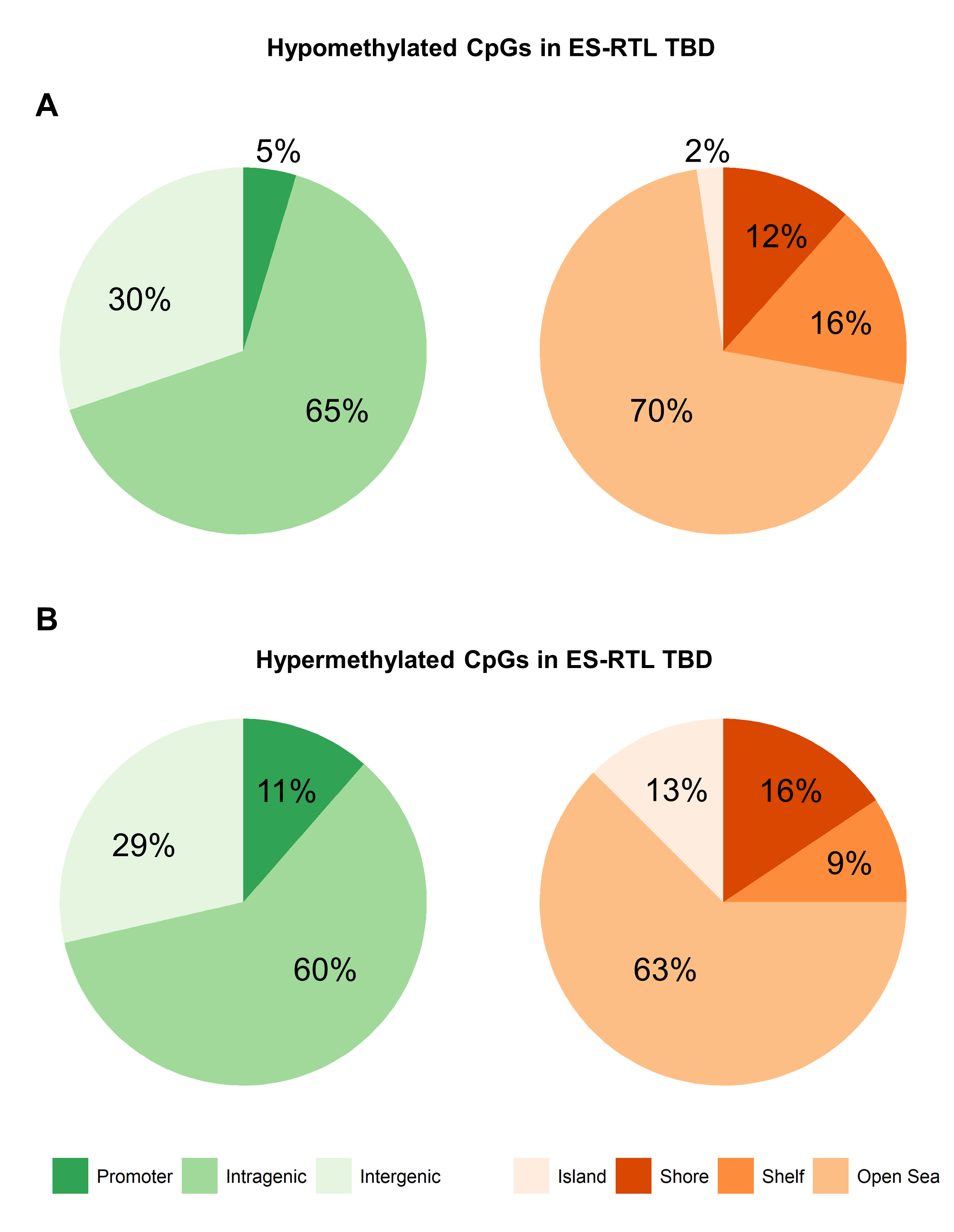

Supplement: Supplementary file 5 — Supplementary Figure 4. [file 41598_2023_34922_MOESM5_ESM.tif]

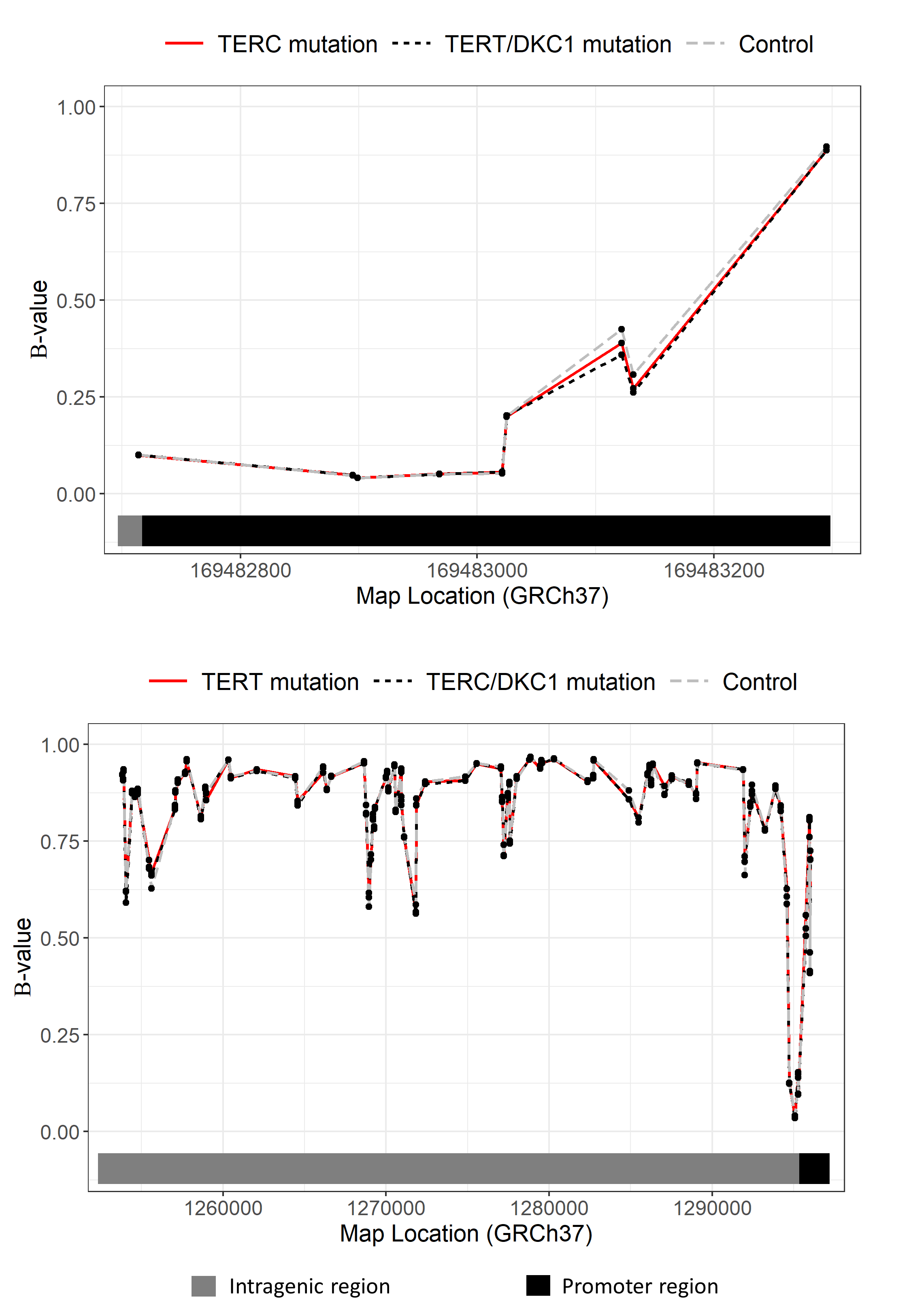

Supplement: Supplementary file 6 — Supplementary Figure 5. [file 41598_2023_34922_MOESM6_ESM.tif]

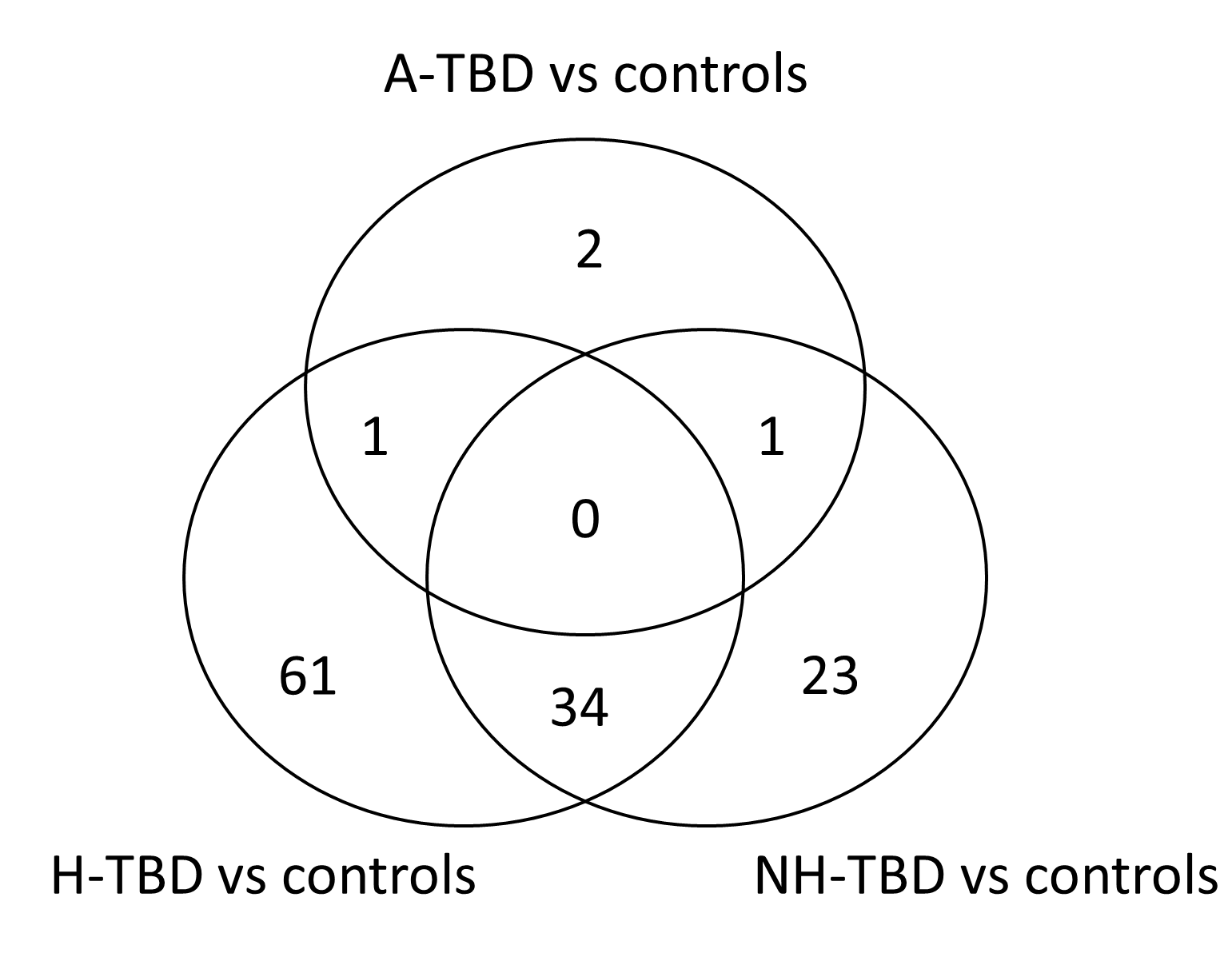

Supplement: Supplementary file 7 — Supplementary Figure 6. [file 41598_2023_34922_MOESM7_ESM.tif]

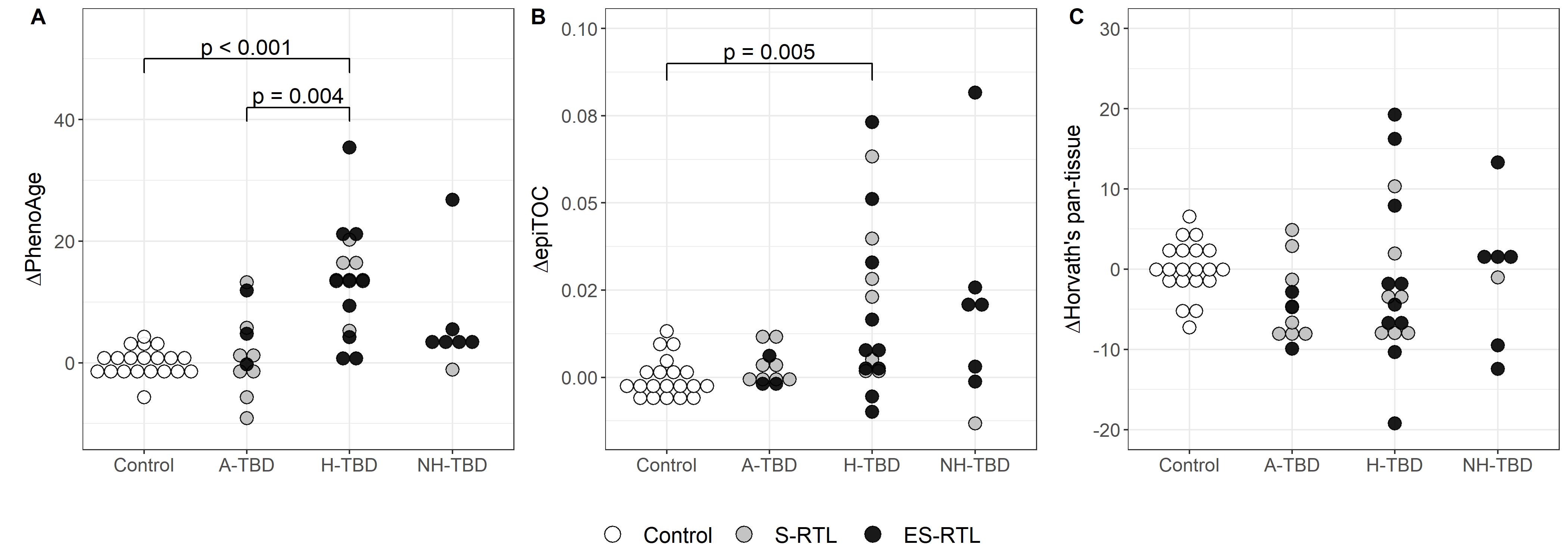

Supplement: Supplementary file 8 — Supplementary Figure 7. [file 41598_2023_34922_MOESM8_ESM.tiff]
